# C1GALT1 is associated with poor survival and promotes soluble Ephrin A1-mediated cell migration through activation of EPHA2 in gastric cancer

**DOI:** 10.1038/s41388-020-1178-7

**Published:** 2020-01-31

**Authors:** Po-Chu Lee, Syue-Ting Chen, Ting-Chun Kuo, Tzu-Chi Lin, Mei-Chun Lin, John Huang, Ji-Shiang Hung, Chia-Lang Hsu, Hsueh-Fen Juan, Po-Huang Lee, Min-Chuan Huang

**Affiliations:** 1 Department of Surgery, National Taiwan University Hospital, Taipei, Taiwan; 2 Department of Traumatology, National Taiwan University Hospital, Taipei, Taiwan; 3 Graduate Institute of Clinical Medicine, College of Medicine, National Taiwan University, Taipei, Taiwan; 4 Graduate Institute of Anatomy and Cell Biology, College of Medicine, National Taiwan University, Taipei, Taiwan; 5 Department of Otolaryngology, National Taiwan University Hospital, Taipei, Taiwan; 6 Department of Medical Research, National Taiwan University Hospital, Taipei, Taiwan; 7 Department of Life Science, National Taiwan University, Taipei, Taiwan; 8 Department of Surgery, E-DA Hospital, Kaohsiung City, Taiwan

**Keywords:** Growth factor signalling, Gastric cancer

## Abstract

C1GALT1 controls the crucial step of GalNAc-type O-glycosylation and is associated with both physiologic and pathologic conditions, including cancers. EPH receptors comprise the largest family of receptor tyrosine kinases (RTKs) and modulate a diverse range of developmental processes and human diseases. However, the role of C1GALT1 in the signaling of EPH receptors remains largely overlooked. Here, we showed that C1GALT1 high expression in gastric adenocarcinomas correlated with adverse clinicopathologic features and is an independent prognostic factor for poor overall survival. Silencing or loss of C1GALT1 inhibited cell viability, migration, invasion, tumor growth and metastasis, as well as increased apoptosis and cytotoxicity of 5-fluorouracil in AGS and MKN45 cells. Phospho-RTK array and western blot analysis showed that C1GALT1 depletion suppressed tyrosine phosphorylation of EPHA2 induced by soluble Ephrin A1-Fc. O-glycans on EPHA2 were modified by C1GALT1 and both S277A and T429A mutants, which are O-glycosites on EPHA2, dramatically enhanced phosphorylation of Y588, suggesting that not only overall O-glycan structures but also site-specific O-glycosylation can regulate EPHA2 activity. Furthermore, depletion of C1GALT1 decreased Ephrin A1-Fc induced migration and reduced Ephrin A1 binding to cell surfaces. The effects of C1GALT1 knockdown or knockout on cell invasiveness in vitro and in vivo were phenocopied by EPHA2 knockdown in gastric cancer cells. These results suggest that C1GALT1 promotes phosphorylation of EPHA2 and enhances soluble Ephrin A1-mediated migration primarily by modifying EPHA2 O-glycosylation. Our study highlights the importance of GalNAc-type O-glycosylation in EPH receptor-regulated diseases and identifies C1GALT1 as a potential therapeutic target for gastric cancer.

## Introduction

Gastric cancer is the third leading cause of cancer-related deaths in the world [1]. Surgery remains the main treatment for operable gastric cancer. For advanced gastric cancer, new therapeutic approaches are currently being researched, such as monoclonal antibodies that target HER2 and immune checkpoint inhibitors, or a combination of these with chemotherapy [2].

Glycosylation is the most common posttranslational modification of proteins, and aberrant glycosylation is often observed in cancers [3]. Mucin-type O-glycosylation modifies proteins in the gastrointestinal tract, and this modification is initiated through the transfer of N-acetylgalactosamine (GalNAc) to serine or threonine residues forming Tn antigen [4]. This reaction is catalyzed by a large group of enzymes named UDP-GalNAc:polypeptide N-acetylgalactosaminyltransferases (GALNTs) [5]. Core 1 β1,3-galatosyltransferase (C1GALT1), with the help of its endoplasmic reticulum chaperone Cosmc, transfers galactose (Gal) to Tn antigen to form Galβ1, 3GalNAc (T-antigen) [6]. T-antigen is then modified by other glycosyltransferases to form complex O-glycans [7].

Mucin-type O-glycosylation regulates multiple biological functions such as apoptosis and angiogenesis of cancer cells [8, 9]. In our previous studies, C1GALT1 promoted the invasive behavior of colon cancer cells [10] and enhanced the proliferation of hepatocellular carcinoma cells [11]. Altered O-glycosylation was reported to influence gastric cancer carcinogenesis cascade and the ensuing cancer progression [12]; however, the underlying mechanisms remain unclear.

Receptor tyrosine kinases (RTKs), such as EGFR, MET, and FGFR2, have been reported to carry O-glycans, and the O-glycosylation can modulate RTK activities [10, 11, 13–15]. Alterations in RTK activities, including EGFR, HER2 (ErbB2, EGFR2), and MET (HGFR), are associated with gastric cancer progression [16–18]. Several lines of evidence have indicated that RTKs actively contribute to gastric oncogenesis and disease progression and are recognized to be the target of cancer therapy [19, 20]. Although C1GALT1 is the critical O-glycosylating enzyme, its expression and role in RTK activities in gastric cancer remain unclear. EPH receptors are the largest family of RTKs. More surprisingly, the functional significance of O-glycosylation for EPH receptors has never been reported.

Human EPH receptors are composed of nigh EPHAs and five EPHBs which preferentially bind to their Ephrin A and Ephrin B ligands, respectively. The Ephrin-EPH system controls a diverse range of developmental processes and pathogenesis of diseases ranging from neuronal disorders to cancer [21]. In human cancers, EPH receptors are frequently overexpressed and contribute to tumor progression [22–25]. Therefore, EPH receptors receive much attention and are attractive drug targets [22, 26, 27]. EPHA2 and its ligand Ephrin A1 are overexpressed in gastric adenocarcinoma and elevated EPHA2 expression is associated with poor survival [28, 29]. EPHA2 has been reported to promote epithelial–mesenchymal transition [30, 31] and silencing of EPHA2 inhibits gastric cancer cell growth and invasion [32]. Although EPHA2 plays a critical role in cancers, how EPHA2 activity is regulated remains unclear.

## Results

### C1GALT1 is overexpressed in gastric cancer

**Fig. 1:**
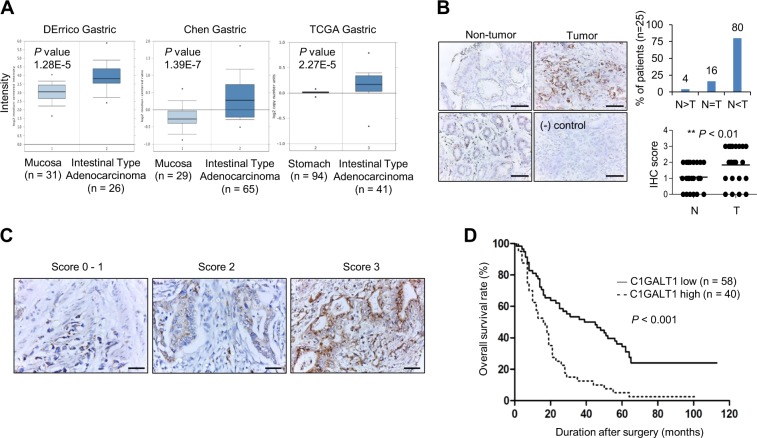
C1GALT1 expression in gastric cancer. **a** *C1GALT1* mRNA expression in normal and cancerous gastric tissues in the Oncomine database. **b** C1GALT1 expression in paired gastric tumors. Immunohistochemical staining revealed C1GALT1 expression in paired gastric adenocarcinoma tumor (right) and nontumor mucosa tissue (left). In nontumor mucosa, foveolar epithelial cells (upper left) expressed less C1GALT1 than glandular epithelial cells (lower left). The negative control (lower right) did not exhibit specific staining. Scale bar, 50 µm. C1GALT1 was frequently overexpressed in gastric adenocarcinoma tumor (T) compared with its surrounding nontumor mucosa (N). *p < 0.05, paired *t* test. **c** Scoring of C1GALT1 expression (0–1, 2, and 3) analyzed using immunohistochemistry. Scale bar, 50 µm. **d** Kaplan–Meier survival analysis according to the expression of C1GALT1 in gastric cancer patients (*n* = 98).

From the Oncomine database, DErrico Gastric, Chen Gastric, and TCGA gastric showed that *C1GALT1* mRNA expression was overexpressed in gastric adenocarcinoma compared with normal gastric mucosa tissue (Fig. 1a). Our immunohistochemical staining indicated that 80% (*n* = 25) of gastric adenocarcinoma tissues showed higher C1GALT1 protein levels than paired nontumor gastric tissues (Fig. 1b). Only 4% of cases exhibited lower C1GALT1 expression in gastric adenocarcinomas. In addition, we observed that glandular epithelial cells in the lower part of nontumor gastric mucosa expressed higher C1GALT1 than surface epithelial cells (Fig. 1b, left panel). These results suggest that C1GALT1 is significantly overexpressed in gastric adenocarcinomas compared with their adjacent nontumor tissues.

### C1GALT1 expression correlates with poor survival in patients with gastric adenocarcinoma

We analyzed the expression of C1GALT1 in gastric cancers from tissue microarray and correlated these with clinicopathologic features and prognoses of gastric cancer. Of the tissue microarray of 111 patients, 13 were excluded because of unqualified slides. The expression of C1GALT1 was scored from 0 to 3 on the basis of the staining intensity of immunohistochemistry (Fig. 1c). Scores 0–2 and 3 were grouped as low and high C1GALT1 expression, respectively. The TNM stage was based on the classification system depending on the invasion of gastric wall (T), the involvement of lymph node (N), and the presence of distant metastasis (M) [33]. Histological grading was based on the Goseki grading system [34]. The results indicated that high C1GALT1 expression correlated with advanced cancer (stages III and IV), higher histological grade, advanced tumor invasion, and nodal metastasis (Table 1). Kaplan–Meier survival curves indicated that high C1GALT1 expression correlated with poor overall survival (*p* < 0.001, Fig. 1d). Univariate analysis indicated that high C1GALT1 expression, advanced cancer stage, higher histological grade, and nodal metastasis significantly correlated with mortality (Table 2). Furthermore, Cox regression analysis revealed that C1GALT1 and advanced cancer stage were independent predictors of mortality. These findings suggest that C1GALT1 is an independent prognostic factor for poor survival in patients with gastric adenocarcinoma.

**Table 1:** Correlation of C1GALT1 intensity and clinicopathologic features.

C1GALT1 intensity	Low (*n* = 58)	High (*n* = 40)	*p* value
Stage, III–IV vs. I–II	1.76 ± 0.75	2.48 ± 0.59	<0.001
Grade, 3 + 4 vs. 1 + 2	2.24 ± 0.78	2.25 ± 0.71	0.937
T, 2–4 vs. 1	1.31 ± 0.48	2.38 ± 0.66	<0.001
N, yes vs. no	1.50 ± 0.67	2.45 ± 0.60	<0.001
M^a^, yes vs. no	2.21 ± 0.74	2.60 ± 0.55	0.252

^a^Nine patients presented with metastatic disease.

**Table 2:** Univariate and multivariate Cox regression analysis for predictors of mortality.

	Univariate		Multivariate	
	HR (95% CI)	*p* value	HR (95% CI)	*p* value
C1GALT1, high vs. low	2.58 (1.64, 4.07)	<0.001	1.94 (1.21, 3.11)	0.006
Stage, III–IV vs. I–II	3.56 (2.16, 5.87)	<0.001	2.85 (1.67, 4.86)	<0.001
Grade, 3 + 4 vs. 1 + 2	1.79 (1.10, 2.92)	0.020		
T^a^, 2–4 vs. 1	NA	NA		
N, yes vs. no	6.51 (3.29, 12.88)	<0.001		
M^b^, yes vs. no	0.93 (0.38, 2.29)	0.876		

*NA* not applicable.

^a^Thirteen patients presented with early gastric cancer, T1 disease.

^b^Nine patients presented with metastatic disease.

### C1GALT1 promotes malignant behaviors of gastric cancer cells

**Fig. 2:**
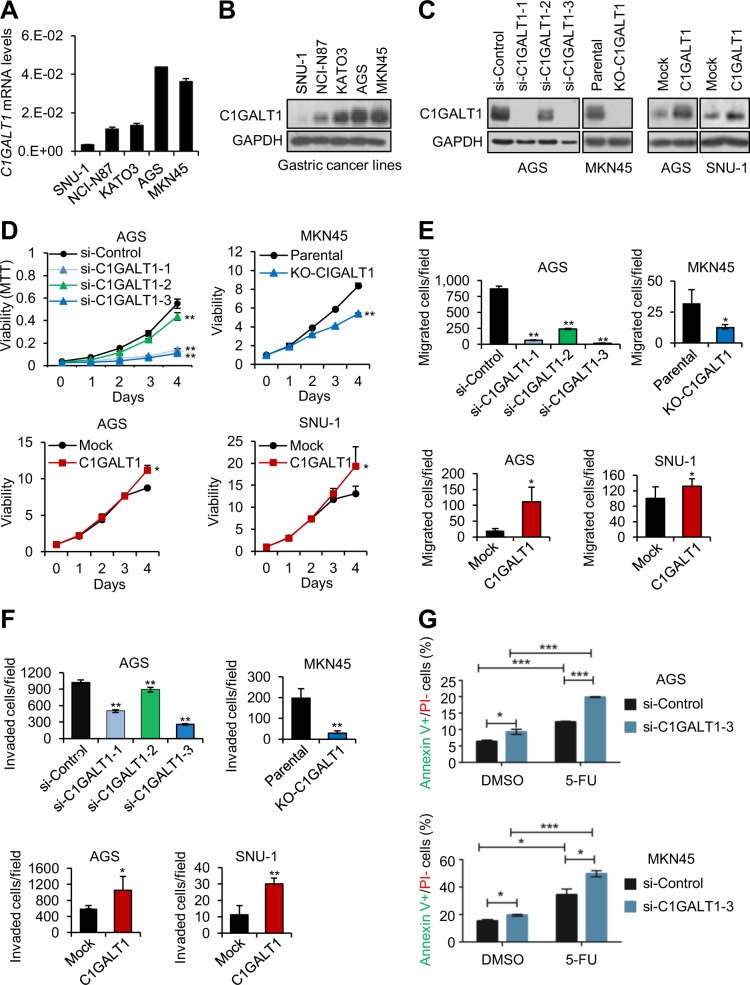
C1GALT1 promotes malignant behaviors of gastric cancer cells. **a** *C1GALT1* expression in gastric cancer cells analyzed by Q-RT-PCR. **b** C1GALT1 expression in gastric cancer cells analyzed by western blot analysis. **c** Western blots showing C1GALT1 knockdown (left panel) or overexpression (right panel) in gastric cancer cells. For knockdown, AGS cells were transfected with nontargeting siRNA (si-Control) or three independent siRNAs against *C1GALT1* (si-C1GALT1-1, si-C1GALT1-2, and si-C1GALT1-3). For C1GALT1 knockout in MKN45 cells, CRISPR/Cas9 system was used. For overexpression, AGS and SNU-1 cells were transfected with empty pcDNA3.1 (Mock) or C1GALT1-pcDNA3.1 (C1GALT1) plasmid. **d** Cell viability was analyzed using MTT assays. C1GALT1 knockdown or knockout decreased cell viability in AGS and MKN45 cells, respectively (upper panel). C1GALT1 overexpression enhanced cell viability in AGS and SNU-1 cells (lower panel). Data are presented as mean (*n* = 3) ± SD. **e** Cell migration was analyzed using transwell migration assays. C1GALT1 knockdown or knockout decreased cell migration in AGS and MKN45 cells, respectively (upper panel). C1GALT1 overexpression enhanced cell migration in AGS and SNU-1 cells (lower panel). Data are presented as mean (*n* = 3) ± SD. **f** Cell invasion was analyzed using Matrigel invasion assays. C1GALT1 knockdown or knockout decreased cell invasion in AGS and MKN45 cells, respectively (upper panel). C1GALT1 overexpression enhanced cell invasion in AGS and SNU-1 cells (lower panel). Data are presented as mean (*n* = 3) ± SD. **g** C1GALT1 knockdown enhanced 5-FU-induced apoptosis in AGS and MKN45 cells. C1GALT1 was knocked known with si-C1GALT1-3 and then treated with solvent DMSO or 5 µg/ml of 5-FU. Apoptosis was analyzed using flow cytometry with FITC-annexin V and PI. Cells were cultured or stimulated with 10% FBS as a chemoattractant. Representative results are shown from at least three independent experiments. Statistical data were analyzed and obtained through Student’s *t* test and graphed as mean ± SD. *p < 0.05; **p < 0.01; ***p < 0.001.

To assess the effect of C1GALT1 on gastric cancer cells, we analyzed cell viability, migration, invasion, and chemoresistance using MTT, transwell migration, Matrigel invasion, and flow cytometry assays, respectively. Q-RT-PCR (Fig. 2a) and western blotting (Fig. 2b) showed variable C1GALT1 expression in five gastric cancer cell lines. C1GALT1 knockdown, knockout, and overexpression in gastric cancer cells were confirmed by western blotting (Fig. 2c). Flow cytometry showed that C1GALT1 knockdown or knockout indeed affected O-glycan expression on the surfaces of AGS and MKN45 cells, as revealed through VVA and PNA staining (Supplementary Fig. S1). Phenotypic assays indicated that C1GALT1 knockdown or knockout significantly suppressed the viability (Fig. 2d), migration (Fig. 2e), and invasion (Fig. 2f) in AGS cells and MKN45 cells, respectively. By contrast, C1GALT1 overexpression enhanced these phenotypes in AGS and SNU-1 cells. Moreover, we observed that si-C1GALT1-2 siRNA with lower C1GALT1 knockdown efficiency exerted a weaker effect on these phenotypes compared with the other two siRNAs. Because si-C1GALT1-1 and si-C1GALT1-3 exhibited excellent knockdown efficiency, we used these two siRNAs for other experiments. Because altered glycosylation has been reported to modulate chemoresistance [35], we examined whether C1GALT1 could regulate 5-FU cytotoxicity in gastric cancer cells. Flow cytometry with FITC-annexin V and PI showed that C1GALT1 knockdown significantly increased apoptosis in both AGS and MKN45 cells compared with control siRNA knockdown cells (Fig. 2g). Taken together, these results suggest that C1GALT1 promotes malignant behaviors of gastric cancer cells.

### Effects of C1GALT1 on tumor growth and metastasis in NOD/SCID mice

**Fig. 3:**
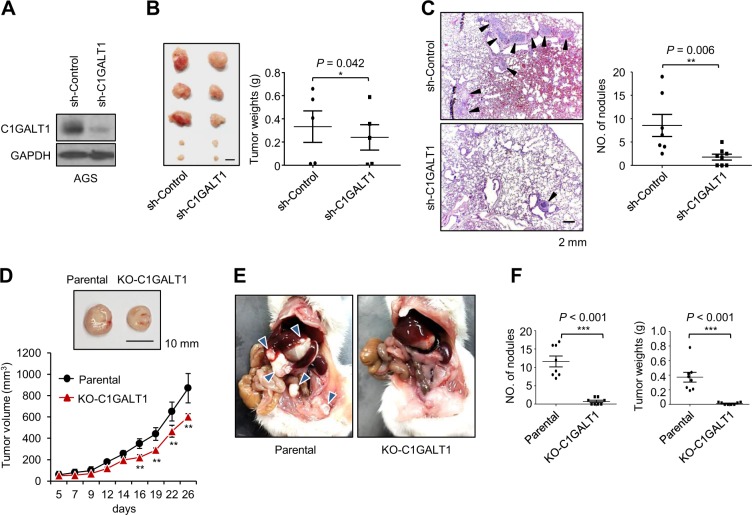
Effects of C1GALT1 on tumor growth and metastasis in NOD/SCID mice. **a** Western blots showing stable C1GALT1 knockdown in AGS cells. **b** C1GALT1 knockdown decreased tumor sizes (upper panel) and weights (lower panel) in NOD/SCID mice subcutaneously injected with AGS cells (*n* = 5 for each group). Mice were sacrificed at day 45. Data are presented as mean ± SEM. This experiment was conducted once. **c** C1GALT1 knockdown decreased lung metastasis in NOD/SCID mice intravenously injected with AGS cells. Mice were sacrificed at day 52. Representative H&E staining images of paraffin-embedded lung sections are shown. Black arrowheads indicate tumor nodules. Results are shown as means ± SD. **p *<* 0.01. This experiment was conducted once. **d** C1GALT1 knockout suppressed tumor growth in a NOD/SCID mouse model. Parental and C1GALT1 knockout (KO-C1GALT1) MKN45 cells were subcutaneously injected into NOD/SCID mice. Mice (*n* = 5 for each group) were sacrificed at day 28. Upper panel, representative image of tumor xenografts was shown. Lower panel, the volume of tumors was measured at different time points. The dimensions of the xenografts were measured using an electronic digital caliper, and the measurements were converted to the xenograft volume (1/2 × length × width^2^). **p < 0.01. This experiment was conducted once. **e** Representative images of tumor formation in NOD/SCID mice intraperitoneally injected with parental or C1GALT1 knockout MKN45 cells. Mice were sacrificed at day 30 (*n* = 8 for each group). Blue arrowheads indicate tumor nodules. **f** Statistical results of NOD/SCID mice intraperitoneally injected with parental or C1GALT1 knockout MKN45 cells. C1GALT1 knockout significantly decreased numbers of tumor nodules (left panel) and total tumor weights (right panel). ***p < 0.001. Data are representative of two independent experiments. All statistical data were analyzed and obtained through Student’s *t* test.

To further investigate the effect of C1GALT1 on gastric tumor growth, C1GALT1 knockdown AGS and C1GALT1 knockout MKN45 cells were xenografted in NOD/SCID mice. We established stable C1GALT1 knockdown AGS cells using short hairpin (sh) RNA and the C1GALT1 knockdown was confirmed by western blotting (Fig. 3a). We found that silencing of C1GALT1 significantly decreased tumor sizes and tumor weights in NOD/SCID mice subcutaneously injected with AGS cells. (Fig. 3b). Next, we analyzed the effect of C1GALT1 on lung metastasis of AGS cells using a mouse tail vein injection model. H&E staining of the lung sections revealed that C1GALT1 knockdown decreased the number of metastatic tumor nodules in the lungs (Fig. 3c). To further confirmed the effect of C1GALT1 on gastric tumor growth, C1GALT1 knockout MKN45 cells were subcutaneously injected into NOD/SCID mice. The results also showed that the loss of C1GALT1 inhibited tumor sizes and tumor growth (Fig. 3d). Moreover, we tested the effect of C1GALT1 knockout on peritoneal tumor growth because peritoneal dissemination is the most frequent metastatic pattern of gastric cancer. Results showed that C1GALT1 knockout significantly decreased the number of MKN45 tumor nodules and total tumor weights in the peritoneal cavity of NOD/SCID mice (Fig. 3e, f). These results suggest that silencing or loss of C1GALT1 inhibits subcutaneous and peritoneal tumor growth of gastric cancer cells in vivo.

### Effects of C1GALT1 knockdown on multiple phospho(p)-RTKs in gastric cancer cells

We have demonstrated that phosphorylation of multiple RTKs, such as EGFR, FGFR2, IGF1R, and MET, can be regulated by O-glycosylation in various cancers [10, 11, 36, 37]. Consistently, we found that C1GALT1 knockdown decreased the level of several p-RTKs using p-RTK array analysis, including EGFR, MET (HGFR), HER2 (ErbB2), Flt-3, Insulin R, IGF-1R, and ROR2 in AGS cells treated with 10% FBS (Supplementary Fig. S2). Western blotting confirmed that C1GALT1 knockdown decreased the phosphorylation of EGFR, HER2, and AKT in both AGS and MKN45 cells (Supplementary Fig. S3A). Because the presence of O-glycans on HER2 and the effect of C1GALT1 on its O-glycans are unclear, we further analyzed HER2 O-glycosylation using VVA and PNA pull-down assays. Our data indicated that VVA lectin could pull down HER2 in parental AGS and MKN45 cells, although the extent was low (Supplementary Fig. S3B), which could be because of low HER2 expression in these cells. Subsequently, we overexpressed HER2 in HEK293FT cells and found that VVA and PNA could also pull down HER2 after benzyl-α-GalNAc treatment (Supplementary Fig. S3B). We used another gastric cancer cell line, N87, which expresses high levels of HER2, and found that C1GALT1 knockdown drastically increased HER2 pulled down through VVA (Supplementary Fig. S3C). C1GALT1 knockdown also decreased the phosphorylation of HER2 in N87 cells. Furthermore, C1GALT1-mediated cell growth was suppressed by lapatinib, a dual inhibitor for EGFR and HER2 (Supplementary Fig. S4). These results suggest that C1GALT1 knockdown modifies O-glycosylation and decreases 10% FBS-mediated phosphorylation of multiple RTKs including EGFR and HER2 in gastric cancer cells.

### Impacts of C1GALT1 on Ephrin A1-triggered phosphorylation of EPHA2

**Fig. 4:**
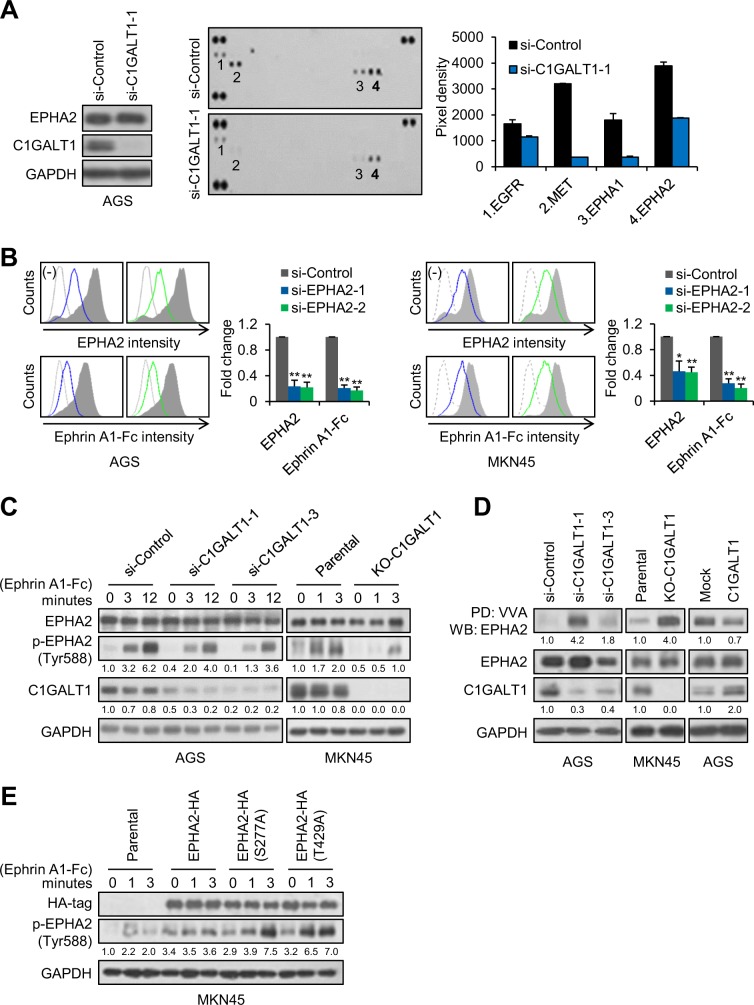
Impacts of O-glycosylation on Ephrin A1-triggered phosphorylation of EPHA2. **a** Phospho-RTK array analysis showing effects of C1GALT1 knockdown on levels of phospho-RTKs in AGS cells treated with Ephrin A1-Fc. Left, western blots showing that AGS cells were knocked down with C1GALT1-specific siRNA. GAPDH was the loading control. Middle, phospho-RTK arrays showing levels of phospho-RTKs in AGS cells transfected with si-Control or si-C1GALT1-1. The cells were serum-starved for 24 h and subsequently treated with 0.2 µg/ml of Ephrin A1-Fc for 3 min. Right, quantification of phospho-RTK levels. **b** EPHA2 was the predominant EPH receptor for Ephrin A1-Fc in AGS (left panel) and MKN45 (right panel) cells. Cells were knocked down by two independent si-RNAs against *EPHA2* and then stained with anti-EPHA2 antibody or Ephrin A1-Fc by flow cytometry. Representative images and their statistical analyses were shown from at least three independent experiments. Data are presented as mean ± SD (*n* = 3). *p *<* 0.05; **p < 0.01. **c** C1GALT1 knockdown or knockout decreased phosphorylation of EPHA2 at Tyr588 in AGS and MKN45 cells. Cells were treated with Ephrin A1-Fc for different times as indicated. Western blots showing expression of EPHA2, p-EPHA2 (Tyr588), C1GALT1, and GAPDH. Relative signal intensities were quantified by ImageJ and shown below the protein bands. Representative results from three independent experiments were shown. **d** C1GALT1 could modify the Tn antigen expression on EPHA2 in AGS and MKN45 cells. EPHA2 in cells was pulled down (PD) using VVA agarose beads and then western blotted (WB) for EPHA2. Expression of EPHA2, C1GALT1, and GAPDH in whole lysates was also shown. Representative results from three independent experiments were shown. **e** Mutations in O-glycosylation sites, S277 and T429, on EPHA2 modulated phosphorylation of EPHA2. S277 and T429 on human EPHA2 were mutated to Ala (A) using site-directed mutagenesis kit. Parental MKN45 cells were transfected with wild-type EPHA2-HA, EPHA2(S277A)-HA mutant, or EPHA2(T429A)-HA mutant. The cells were treated with Ephrin A1-Fc for different times as indicated. Western blots showing ectopic expression of HA-tagged EPHA2, p-EPHA2 (Tyr588), and GAPDH. Representative results from three independent experiments were shown.

Surprisingly, the effect of O-glycosylation on EPH receptors, the largest family of RTKs, has never been reported. Ephrin A1 is the ligand for EPHA receptors and is overexpressed in gastric cancer. In addition, soluble Ephrin A1 has been reported to be secreted in conditioned media of several cancer cell lines and be detected in serum of cancer patients [38–40]. We found that soluble Ephrin A1 was present in conditioned media of AGS and MKN cells (Supplementary Fig. S5). To systematically examine whether C1GALT1-mediated O-glycosylation can regulate the activity of the EPH receptor family, soluble Ephrin A1-Fc was used to treat AGS cells transfected with control siRNA or C1GALT1 siRNA and then p-RTK array analysis was performed. Interestingly, C1GALT1 knockdown decreased levels of p-EPHA1 and p-EPHA2 as well as p-EGFR and p-MET (Fig. 4a). Using short exposure, EPHA2 was the first activated by Ephrin A1-Fc. To know whether decreased p-EGFR and p-MET were mediated by Ephrin A1-Fc treatment, AGS cells transfected with control or C1GALT1-specific siRNA were serum starved and then treated with or without Ephrin A1-Fc. The results showed that p-EGFR and p-MET were decreased by C1GALT1 siRNA even without Ephrin A1-Fc treatment (Supplementary Fig. S6), suggesting that the decrease in p-EGFR and p-MET in the p-RTK array does not depend on Ephrin A1-mediated signaling. However, we noticed that Ephrin A1-Fc could slightly increase phosphorylation of EGFR but not MET. To examine whether EPHA2 was the major EPHA receptor for Ephrin A1, we knocked down EPHA2 with two independent siRNAs and analyzed the Ephrin A1-Fc binding to AGS and MKN45 cells. Flow cytometry showed that EPHA2 knockdown decreased EPHA2 levels by 76.6%–78.2% and 53.8%–55.2% in AGS and MKN45 cells, respectively, and inhibited Ephrin A1 binding by 79.5%–82.9% and 72.2%–79.7%, respectively (Fig. 4b), indicating that EPHA2 is the predominant EPH receptor for Ephrin A1-Fc in gastric cancer cells. Western blot analysis validated that C1GALT1 knockdown and knockout decreased Ephrin A1-Fc induced phosphorylation of EPHA2 at Y588 in AGS and MKN45 cells, respectively (Fig. 4c). Next, we performed VVA lectin pull-down assays to examine whether EPHA2 was decorated with O-glycans and whether C1GALT1 could modify the O-glycans. We found that EPHA2 could be pulled down with VVA and the levels were enhanced by C1GALT1 knockdown and knockout in AGS and MKN45 cells, respectively (Fig. 4d). By contrast, C1GALT1 overexpression in AGS cells decreased the amounts of EPHA2 pulled down by VVA.

To rule out the possibility that the VVA binding to EPHA2 is dependent on N-glycans, we further used PNGaseF to remove N-glycans in lysates and then performed VVA pull-down assays. Results showed that C1GALT1 knockdown still increased the amounts of EPHA2 pulled down by VVA after removal of N-glycans (Supplementary Fig. S7). These findings suggest that C1GALT1 can modify O-glycans on EPHA2 and regulate soluble Ephrin A1-induced tyrosine phosphorylation of EPHA2.

Henrik Clausen’s team identified two O-glycosites, S277 and T429, on EPHA2 in gastric cancer cells using proteomics approaches [41]. To further determine whether site-specific O-glycosylation can modulate EPHA2 activity, we constructed S277A and T429A mutants using site-directed mutagenesis. Interestingly, our results showed that both S277A and T429A mutants dramatically enhanced phosphorylation of Y588 (Fig. 4e). These results suggest that not only overall O-glycan structures on EPHA2 but also site-specific O-glycosylation can regulate EPHA2 activity.

### Effects of EPHA2 knockdown on gastric cancer cells in vitro and in vivo

**Fig. 5:**
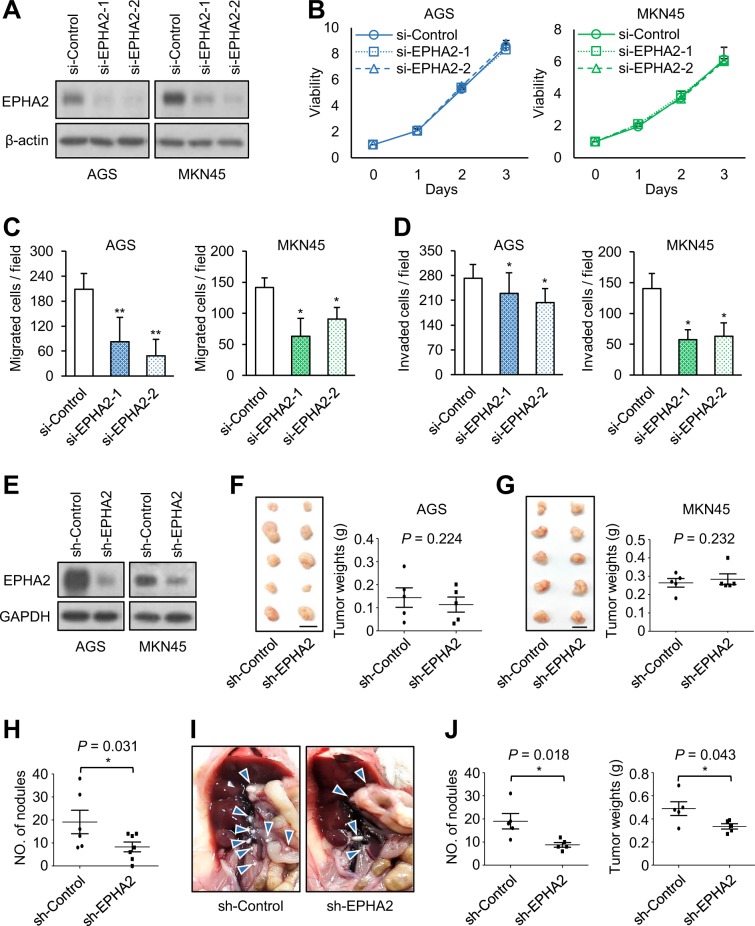
Effects of EPHA2 knockdown on gastric cancer cells in vitro and in vivo. **a** Western blots showing EPHA2 knockdown in AGS and MKN45 cells. **b** Cell viability was not significantly affected by EPHA2 knockdown in AGS and MKN45 cells analyzed by MTT assays. Cells were transfected with two independent siRNAs (si-EPHA2-1 and si-EPHA2-2) or nontargeting control siRNA (si-Control). Data are presented as mean (*n* = 3) ± SD. **c** Cell migration was analyzed using transwell migration assays. Data are presented as mean (*n* = 3) ± SD. *p < 0.05; **p < 0.01. **d** Cell invasion was analyzed using Matrigel invasion assays. Data are presented as mean (*n* = 3) ± SD. *p < 0.05. **e** Western blots showing stable EPHA2 knockdown in AGS and MKN45 cells. **f** Tumor sizes (left panel) and weights (right panel) were not significantly affected by EPHA2 knockdown in NOD/SCID mice subcutaneously injected with AGS cells. Mice were sacrificed at day 45 (*n* = 5 for each group). This experiment was conducted once. **g** Tumor sizes (left panel) and weights (right panel) were not significantly affected by EPHA2 knockdown in NOD/SCID mice subcutaneously injected with MKN45 cells. Mice were sacrificed at day 30. (*n* = 5 for each group) This experiment was conducted once. **h** EPHA2 knockdown decreased lung metastasis in NOD/SCID mice intravenously injected with AGS cells. Numbers of tumor nodules in lungs are shown as means ± SD. *p *<* 0.05. This experiment was conducted once. **i** Representative images of tumor formation in NOD/SCID mice intraperitoneally injected with MKN45 cells. Mice were sacrificed at day 30. Blue arrowheads indicate tumor nodules. **j** Statistical results of NOD/SCID mice intraperitoneally injected with MKN45 cells. EPHA2 knockdown significantly decreased numbers of tumor nodules (left panel) and total tumor weights (right panel). *p < 0.05. This experiment was conducted once. All statistical data were analyzed and obtained through Student’s *t* test.

To know the in vitro effects of EPHA2 knockdown on gastric cancer cells, we analyzed cell viability, migration, and invasion using MTT, transwell migration, and Matrigel invasion assays, respectively. EPHA2 was knocked down with two independent siRNAs against *EPHA2* in AGS and MKN45 cells, which was confirmed by western blotting (Fig. 5a). Results showed that EPHA2 knockdown did not significantly affect cell viability (Fig. 5b). By contrast, EPHA2 knockdown suppressed cell migration (Fig. 5c) and invasion (Fig. 5d). To assess the in vivo effect of EPHA2 on gastric cancer cells, EPHA2 knockdown AGS and MKN45 cells were xenografted in NOD/SCID mice via subcutaneous or peritoneal injection. The stable knockdown of EPHA2 with shRNA in AGS and MKN45 cells was confirmed by western blotting (Fig. 5e). We found that EPHA2 knockdown did not significantly affect tumor sizes and tumor weights in NOD/SCID mice subcutaneously injected with AGS (Fig. 5f) or MKN45 cells (Fig. 5g). By contrast, in the tail vein injection model, EPHA2 knockdown reduced the number of lung metastatic nodules of AGS cells (Fig. 5h). Moreover, in the peritoneal injection model, EPHA2 knockdown significantly decreased the number of MKN45 tumor nodules and total tumor weights in the peritoneal cavity of NOD/SCID mice (Fig. 5i, j). These results suggest that EPHA2 knockdown inhibits invasive behaviors of gastric cancer cells in vitro and in vivo.

### C1GALT1 mediates its pro-migratory effect on gastric cancer cells through EPHA2

**Fig. 6:**
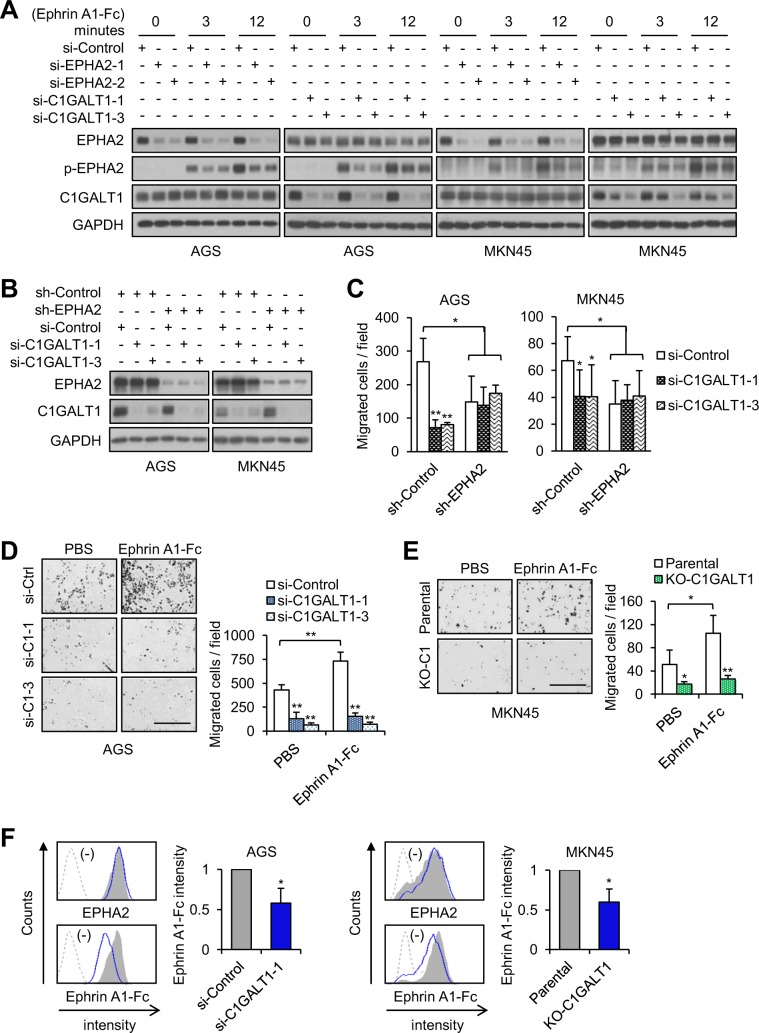
C1GALT1 mediates its pro-migratory effect on gastric cancer cells through EPHA2. **a** Both C1GALT1 knockdown and EPHA2 knockdown decreased phosphorylation of EPHA2 at Tyr588 in AGS and MKN45 cells. EPHA2 and C1GALT1 were knocked known with siRNAs, as indicated, in AGS and MKN45 cells. The cells were treated with Ephrin A1-Fc for different times as indicated. Western blots showing expression of EPHA2, p-EPHA2 (Tyr588), C1GALT1, and GAPDH. Representative results from three independent experiments were shown. **b** Western blots showing siRNA-mediated C1GALT1 knockdown in stable EPHA2 knockdown AGS and MKN45 cells. **c** Cell migration analyzed using transwell migration assays. AGS and MKN45 cells were seeded in Boyden chambers with 10% FBS as a chemoattractant. Student’s *t* test and graphed as mean ± SD (*n* = 3). *p < 0.05; **p < 0.01. **d** C1GALT1 knockdown inhibited Ephrin A1-Fc-triggered migration in AGS cells. Cells were pretreated with Ephrin A1-Fc and then subjected to transwell migration assays using 10% FBS as a chemoattractant. Representative images of migrated cells (left panel) and statistical result were shown. Scale bars indicate 1 mm. Student’s *t* test and graphed as mean ± SD (*n* = 3). *p < 0.05; **p < 0.01. **e** C1GALT1 knockout inhibited Ephrin A1-Fc-triggered migration in MKN45 cells. Cells were pretreated with Ephrin A1-Fc and then subjected to transwell migration assays using 10% FBS as a chemoattractant. Representative images of migrated cells (left panel) and statistical result were shown. Scale bars indicate 1 mm. Student’s *t* test and graphed as mean ± SD (*n* = 3). *p < 0.05; **p < 0.01. **f** C1GALT1 knockdown and C1GALT1 knockout decreased binding of Ephrin A1-Fc to AGS and MKN45 cells, respectively. Cells were stained with anti-EPHA2 antibody or Ephrin A1-Fc and then analyzed by flow cytometry. Bar diagrams showing relative Ephrin A1-Fc binding to cells. Data are presented as mean ± SD (*n* = 3). *p < 0.05 using student’s *t* test.

Soluble Ephrin A1 can promote metastasis in several cancer types [38, 39, 42]. We found that C1GALT1 modified O-glycans on EPHA2 and regulated EPHA2 phosphorylation. In addition, the effects of C1GALT1 knockdown on cell invasiveness in vitro and in vivo were phenocopied by EPHA2 knockdown in gastric cancer cells. To further confirm that C1GALT1 mediated its pro-migratory effect at least partly through EPHA2, we analyzed EPHA2 phosphorylation and cell migration in C1GALT1 knockdown, EPHA2 knockdown, or double knockdown gastric cancer cells. Similar as EPHA2 knockdown, C1GALT1 knockdown also decreased Ephrin A1-Fc induced phosphorylation of EPHA2 at Y588 in both AGS and MKN45 cells (Fig. 6a, Supplementary Fig. S8). It has been reported that Ephrin A1 can induce phosphorylation of STAT3, AKT, ERK, FAK, or Src in several cell types [43, 44]. Therefore, we analyzed these molecules in gastric cancer cells. We found that, among them, only ERK phosphorylation was induced by Ephrin A1-Fc in AGS cells. Furthermore, both C1GALT1 knockdown and EPHA2 knockdown exerted an inhibitory effect on ERK phosphorylation in AGS cells (Supplementary Fig. S9). No significant changes in the phosphorylation of other molecules were observed. Although the downstream signaling pathways of Ephrin A1-EPHA2 in gastric cancer cells remain unclear, the effects of C1GALT1 and EPHA2 on phosphorylation of these molecules, including EPHA2, STAT3, AKT, ERK, FAK, and Src, were similar.

To investigate whether the effect of C1GALT1 siRNAs on cell migration was reduced in EPHA2 knockdown cells, stable EPHA2 knockdown AGS and MKN cells were transiently transfected with two independent C1GALT1 siRNAs. EPHA2 and/or C1GALT1 knockdown were confirmed by western blotting (Fig. 6b). Transwell migration assays showed that C1GALT1 knockdown or EPHA2 knockdown significantly inhibited cell migration in both AGS and MKN45 cells (Fig. 6c). However, in EPHA2 knockdown cells, C1GALT1 siRNAs were unable to decrease cell migration (Fig. 6c). To investigate whether Ephrin A1-Fc-induced migration could be suppressed by C1GALT1 knockdown or C1GALT1 knockout in gastric cancer cells, cell migration of AGS and MKN45 cells stimulated with Ephrin A1-Fc was analyzed. Results of transwell migration assays showed that Ephrin A1-Fc increased migration of AGS (Fig. 6d) and MKN45 cells (Fig. 6e). We confirmed that the Fc of human IgG did not affect cell migration (Supplementary Fig. S10). Moreover, C1GALT1 knockdown or knockout significantly inhibited Ephrin A1-Fc mediated migration and blocked the effect of Ephrin A1-Fc on AGS (Fig. 6d) and MKN45 cells (Fig. 6e), respectively. Next, we examined whether binding of Ephrin A1-Fc to surfaces of gastric cancer cells was affected by C1GALT1. Results from flow cytometry showed that C1GALT1 knockdown or knockout significantly decreased Ephrin A1-Fc binding to AGS and MKN45 cells, respectively (Fig. 6f). These results suggest that C1GALT1 mediates its pro-migratory effect on gastric cancer cells at least partly through EPHA2.

### Functional pathways affected by C1GALT1 knockdown

**Fig. 7:**
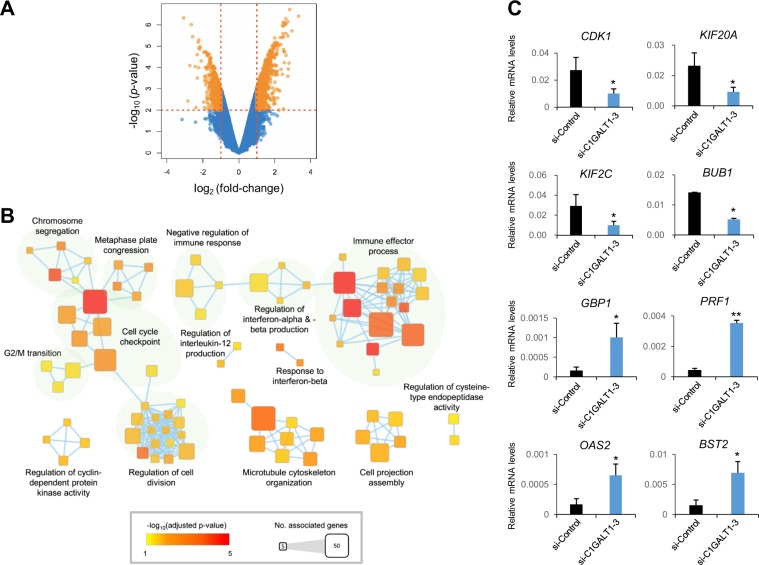
cDNA microarray analysis. **a** Volcano plot analysis of microarray showing that 1491 probes in the C1GALT1 knockdown AGS cells were significantly (*p* < 0.01) altered by twofold or more versus the control. Orange areas indicate significant changes in gene expression. **b** Functional map of differentially expressed genes. The over-representative GO terms of *C1GALT1* knockdown AGS cells were mapped as networks. A node and each edge represent each enriched gene set (*p* < 0.05) and the gene overlap score between nodes passing a threshold (0.6), respectively. Node color encodes the enrichment *p* value (red: low; yellow: high). The node size is proportional to the number of genes belonging to the corresponding gene set. The edge thickness is proportional to the overlap score. Groups of functionally related gene sets are highlighted in colors and as labeled. **c** Quantitative RT-PCR validation of the microarray results. Results are the mean ± SD of three independent experiments. *p < 0.05; **p < 0.01 using Student’s *t* test.

To better understand the mechanism by which C1GALT1 regulates gastric adenocarcinoma progression, we evaluated global gene expression in control and C1GALT1 knockdown AGS cells. Volcano plot analysis revealed 1491 probes in C1GALT1 knockdown AGS cells that were significantly altered by twofold or more versus the control (Fig. 7a). Functional enrichment and network analysis showed that C1GALT1 knockdown in AGS cells affected several functional pathways, including cell cycle checkpoint, immune effector process, microtubule cytoskeleton organization, and regulation of cell division (Fig. 7b). The differential gene expression of the microarray results was validated through quantitative RT-PCR (Fig. 7c and Supplementary Table S2). To validate the functional pathway of microarray data affected by C1GALT1, we analyzed cell cycle through flow cytometry. C1GALT1 knockdown indeed affected the cell cycle (Supplementary Fig. S11). Consistent with our in vitro and in vivo data, these results suggest that C1GALT1 regulates the expression of genes related to several cancer malignant behaviors in gastric cancer cells.

## Discussion

In this study, we found that C1GALT1 was significantly overexpressed in gastric adenocarcinomas; and that C1GALT1 high expression correlated with adverse clinicopathologic features and is an independent prognostic factor for poor overall survival. Knockdown or knockout of C1GALT1 inhibited malignant properties of gastric cancer cells in vitro and suppressed subcutaneous and peritoneal tumor growth in vivo. Importantly, we identified that O-glycosylation is a critical regulatory factor for EPHA2 activity and signaling. Silencing of C1GALT1 decreased tyrosine phosphorylation of EPHA2, inhibited binding of Ephrin A1 to cell surfaces, and suppressed soluble Ephrin A1-induced migration in gastric cancer cells. We also found that the effect of C1GALT1 knockout on cell invasiveness and peritoneal tumor growth of gastric cancer cells was phenocopied by EPHA2 knockdown. These results suggest that C1GALT1 promotes gastric cancer cell invasiveness at least partly through activation of EPHA2. Our findings open novel insights into the role of O-glycosylation in EPHA2 functions and highlight C1GALT1 as a potential diagnostic and therapeutic target for gastric cancer.

RTK signaling pathways are crucial for malignant transformation of cancers [45]. Genomic alterations in RTKs, including EGFR, HER2, FGFR2, and MET, were reported to occur in ~37% of gastric cancer patients [46]. At present, anti-HER2 therapy plays a crucial role in gastric cancer treatment [18]. We showed that silencing of C1GALT1 decreases O-glycosylation and phosphorylation of EGFR and HER2. Moreover, C1GALT1-mediated cell viability was significantly suppressed by lapatinib. These results suggest that C1GALT1 promotes cell viability at least partly through the activation of EGFR and HER2, although other pathways are involved.

EPH receptors constitute the largest family of RTKs and control a diverse range of developmental processes [21]. However, the role of GalNAc-type O-glycosylation in EPH receptors has been largely ignored. We therefore focused on this issue. Our data showed that C1GALT1 depletion increased, whereas C1GALT1 overexpression decreased VVA binding to EPHA2. C1GALT1 knockdown or knockout decreased soluble Ephrin A1-mediated phosphorylation of Y588 on EPHA2. These results suggest that EPHA2 carries GalNAc-type O-glycans and altered O-glycan structures can regulate EPHA2 activity. Our data showed that EPHA2 is the major receptor for Ephrin A1 and silencing C1GALT1 significantly decreases Ephrin A1-Fc binding to the cells. Importantly, the effects of C1GALT1 knockout on cell migration, invasion, and peritoneal tumor growth were phenocopied by EPHA2 knockdown. These results suggest that silencing of C1GALT1 modifies O-glycan structures on EPHA2 to decrease Ephrin A1-Fc binding to EPHA2 and in turn suppresses cell migration.

Henrik Clausen’s team identified O-glycosites at S277 and T429 of EPHA2 using proteomics approaches [41]. To further determine the role of site-specific O-glycosylation in EPHA2 phosphorylation, we mutated serine or threonine to alanine to block the initiation of O-glycosylation. The results showed that lack of O-glycosylation on either S277 or T429 dramatically enhanced phosphorylation of Y588 on EPHA2, suggesting that site-specific O-glycosylation can also determine EPHA2 activity. It will be of great interest to further investigate which GalNAc transferases (GALNTs) initiate O-glycosylation on specific O-glycosites to regulate EPHA2 functions. Our data showed that C1GALT1 knockdown also decreased phospho-EPHA1 levels in AGS cells treated with Ephrin A1-Fc. Henrik Clausen’s glycoproteomic data indicated that EPHB4 is decorated with GalNAc-type O-glycans [41]. Therefore, it is possible that several EPH receptors are O-glycosylated and their functions can be modulated by O-glycosylation. In addition to EPHA2 and EPHA1, we found that soluble Ephrin A1-Fc can activate EGFR, but not MET. Consistently, it has been reported that progranulin activates EPHA2 and concurrently enhances EGFR signaling [47]. These findings support that EPHA2 can crosstalk with other RTKs, such as EGFR, to mediate its functions and imply that targeting EPHA2 could be beneficial for other RTK-dependent diseases, especially cancers.

Although the effectiveness of 5-FU monotherapy is limited due to drug resistance, it is still used as the standard first-line chemotherapy for metastatic gastric cancer [48]. In a previous study, glycosylation was highly involved in the acquisition of multidrug resistance at specific positions and through its changes in secreted glycoproteins [35]. We previously indicated that C1GALT1 knockdown suppresses the MUC1-C/β-catenin signaling pathway in breast cancer cells [49]. Blocking MUC1-C function was revealed to be effective when combined with taxol and doxorubicin to treat breast cancer [50]. Our data consistently provide evidence that silencing C1GALT1 enhances the cytotoxicity of the chemotherapeutic drug 5-FU in gastric cancer cells through potentiating the apoptotic death response. These findings suggest that the combination of C1GALT1 inhibition and 5-FU may be a promising therapy strategy to overcome the resistance of 5-FU in gastric cancer treatment.

In our study, a functional enrichment and network analysis indicated that C1GALT1 regulate several functional pathways. Our results from the clinical analyses and in vitro phenotypic assays support that these functional pathways can be regulated by C1GALT1. For example, the in vitro finding that C1GALT1 knockdown affects cell cycle and cell viability is consistent with the function map showing altered cell cycle checkpoints and cell divisions. C1GALT1 knockdown inhibits gastric cancer cell migration and invasion, which is associated with altered microtubule cytoskeleton organization. Noteworthily, the microtubule organization was also indicated to be related to lymph node metastasis, tumor stage, and poor outcomes of patients with gastric cancer [51], which are associated with C1GALT1 high expression. Microarray data suggested that C1GALT1 modulates genes associated with the immune effector process. Because the targeting of immune checkpoints seems promising in the treatment of several cancers [2], the regulatory role of C1GALT1 in immune responses warrants further investigation.

## Materials and methods

### Patient samples

Twenty-five patients who had undergone surgery at National Taiwan University Hospital were selected for this study. Written consent was obtained from the patients; the hospital’s Institutional Review Board approved this study (IRB No: 201604068RIND). Subsequently, paraffin-embedded tissue blocks of gastric adenocarcinomas and their surrounding nontumor tissues were collected.

### Immunohistochemistry

Tissue microarray of gastric adenocarcinoma from 111 patients was purchased (HStm-Ade178Sur-01, US Biomax, Inc., MD, USA) for immunohistochemical staining. The tissue microarray was incubated with an anti-C1GALT1 antibody (1:100, Santa Cruz Biotechnology, CA, USA) at 4 °C for 16 h. Super Sensitive^TM^ Link-Label IHC Detection System (BioGenex, CA, USA) was used and signals were visualized through a 3,3-diaminobenzidine (DAB) liquid substrate system (Sigma, MO, USA). The tissues were counterstained with hematoxylin and mounted with UltraKitt (J.T. Baker, Deventer, Holland). Negative controls were performed through replacing the primary antibody with a control IgG at the same concentration.

### Cell lines and cell culture

The human gastric cancer cell lines AGS and MKN45 were a gift from Dr Chiung-Nien Chen (National Taiwan University Hospital, Taiwan). AGS and MKN45 cell lines were authenticated using short tandem repeat (STR) profiling analysis. Gastric cancer cell lines NCI-N87, SNU-1, and KATO3 were a gift from Dr I-Rue Lai (National Taiwan University Hospital, Taiwan), who recently purchased them from Bioresources Collection & Research Center (Hsinchu, Taiwan). HEK293FT cells were purchased from Thermo Fisher Scientific. Cell lines were maintained in complete medium containing 10% fetal bovine serum (FBS) (Life Technologies, Burlington, Canada) and 1% penicillin/streptomycin (P/S; Gibco, Invitrogen™, Thermo Fisher Scientific), and cultured at 37 °C in air with 5% CO_2_.

Dulbecco’s Modified Eagle Medium (DMEM) (Thermo Fisher Scientific, Yokohama, Japan) was used for HEK293FT cells. RPMI 1640 medium (GE healthcare, Chicago, USA) was used for AGS, MKN45, NCI-N87, KATO3, and SNU-1 cells.

### cDNA synthesis and real-time RT-PCR

Total RNA was isolated using TRIzol reagent (Invitrogen) according to the manufacturer’s protocol. Two micrograms of total RNA were used in a 20 μL reverse transcription reaction using High-Capacity cDNA Reverse Transcription Kits (AB, USC) for cDNA synthesis. For real-time PCR, QuantStudio 3 Real-Time PCR system (Thermo) was used. The real-time PCR reactions were performed in 20 μL volume containing 1 μL cDNA, 10 μL SensiFAST SYBER Lo-ROX Mix (BIOLINE), and primer pairs. The following primer pairs were used: *C1GALT1*, *CDK1*, *K1F20A*, *F1F2C*, *BUB1*, *GBP1*, *PRF1*, *OAS2*, *BST2*, and *GAPDH* (Supplementary Table S1).

### Transfection and plasmid construction

For transient knockdown, three independent C1GALT1-specific siRNAs (Invitrogen), two independent EPHA2-specific siRNAs (Dharmacon), and nontargeting siRNA (Invitrogen; Dharmacon), were used to transfect gastric cancer cells through Lipofectamine RNAiMAX (Invitrogen) with a final concentration of 10 nM for two days. The siRNAs against C1GALT1 were si-C1GALT1-1: 5′-UUAGUAUACGUUCAGGUAAGGUAGG-3′, si-C1GALT1-2: 5′-UUAUGUUGGCUAGAAUCUGCAUUGA-3′, and si-C1GALT1-3: 5′-CCUACCUUACCUGAACGUAUACUAA-3′. The siRNAs against EPHA2 were si-EPHA2-1: 5′-UGAAUGACAUGCCGAUCUA-3′, and si-EPHA2-2: 5′-GAAGUUCACUACCGAGAUC-3′. The nontargeting siRNAs (si-Control) were 5′-CAACCUCAGCCAUGUCGACUGGUUU-3′. For stable knockdown, shC1GALT1/pLKO (TRCN35411), shEPHA2/pLKO (TRCN6403), and nontargeting control (TRC025) were obtained from National RNAi Core Facility (Academia Sinica, Taipei, Taiwan). For stable overexpression, C1GALT1/pcDNA3.1A [11] or control empty pcDNA3.1A plasmid were used to transfect AGS cells using Lipofectamine 3000 (Invitrogen) according to the manufacturer’s protocol.

### Western blot analysis

Proteins were separated on an 8% SDS-PAGE and transferred onto a PVDF membrane. After blocking with 5% bovine serum albumin (BSA; Bio-Rad, CA, USA) for 1 h at room temperature, membranes were incubated with primary antibodies at 4 °C overnight. Antibodies against C1GALT1, GAPDH, Ephrin A1, and FAK were purchased from Santa Cruz Biotechnology. Antibodies against EGFR, p-EGFR, HER2, p-HER2, p-AKT, EPHA2, p-EPHA2, p-ERK, ERK, p-STAT3, STAT3, and p-FAK were purchased from Cell Signaling Technology (MA, USA). Antibodies against AKT, p-Src, and Src were purchased from GeneTex Inc. (CA, USA). The membranes were then incubated with horseradish peroxidase-conjugated secondary antibodies, and proteins were detected using ECL reagents (GE Healthcare Life Sciences).

### MTT assay

Gastric cancer cells (1.5 × 10^3^) in 100 μL of complete RPMI were seeded in 96-well plates for 16 h; subsequently, 10 μL of 5 mg/mL 3-(4,5-dimethyl-2-thiazolyl)-2,5-diphenyl-2H-tetrazolium bromide solution (MTT; Sigma) was added to each well for the indicated times and incubated at 37 °C for 3 h, and the MTT formazan crystals were dissolved with 100 μL 10% SDS containing 0.01 N HCl. The resultant optical density was measured spectrophotometrically at the dual wavelengths of 550 and 630 nm.

### Transwell migration and Matrigel invasion assays

Cell migration and invasion assays were evaluated using transwell (Corning, NY, USA) or Matrigel-coated (BD Biosciences, CA, USA) transwell chamber, respectively. Each transwell chamber contained a membrane of pore size 8 μm. AGS (3 × 10^4^), MKN45 and SNU-1 cells (2 × 10^5^) in 0.25 mL serum-free RPMI were seeded into the transwell or Matrigel-coated transwell chamber, following which the chambers were put into 24-well plates. After incubating for 24 h or 48 h, the cells were fixed and stained with 0.5% (w/v) crystal violet (Sigma) containing 20% (v/v) methanol. The migrated and invaded cells from three random fields were counted under a microscope.

### Phospho-receptor tyrosine kinase array assay

A human phospho-RTK array kit including 49 RTKs was purchased from R&D Systems (MN, USA). AGS cells were serum-starved for 24 h and stimulated with 10% FBS for 10 min or Ephrin A1-Fc (R&D, MN, USA) for 3 min. Cells were lysed, and 500 μg of protein lysates were subjected to western blotting according to the manufacturer’s protocol.

### Lectin pull-down assay

To analyze changes in O-glycans, 300 μg of total cell lysates were treated with or without neuraminidase (Sigma) to remove sialic acids and then incubated overnight with *Vicia Villosa* lectin (VVA) or peanut agglutinin (PNA) agarose beads (Vector Laboratories, CA, USA) at 4 °C. After washing with PBS twice, the pulled-down proteins were subjected to western blot analysis.

### Flow cytometry

Cells (1 × 10^5^) were resuspended in 100 μL PBS with 1% BSA and then stained with VVA-FITC, PNA-FITC (Vector Laboratories), Ephrin A1-Fc, or anti-EPHA2 primary antibody (R&D, MN, USA) on ice for 30 min. FITC-conjugated secondary antibody was used. After washing twice, the fluorescence intensity was analyzed using a flow cytometer (BD LSR-II; BD Pharmingen). For apoptosis assay, cells were treated with DMSO solvent or 5-fluorouracil (5-FU) and stained with annexin V-FITC and propidium iodide (PI). Samples not containing the fluorescent reagents were used as negative controls.

### cDNA microarray analysis

To determine the C1GALT1-regulated gene expression profile in AGS cells, C1GALT1 was knocked down using *C1GALT1*-specific siRNA (Invitrogen™, Thermo Fisher Scientific) in AGS cells, and nontargeting siRNA was used as the control. Total RNA was extracted from AGS cells using a GeneJET RNA Purification Kit (Thermo Scientific) and was subjected to cDNA microarray analysis (Agilent-072363 SurePrint G3 Human GE v3 8 × 60 K Microarray 039494) and gene ontology enrichment analysis as described previously [15]. The selected genes with differential expression were validated through real-time RT-PCR analysis. Microarray data were deposited in the GEO database (accession number GSE90672).

The raw data are normalized using the quantile method and the differential expressed genes were determined using the R package ‘limma’. The over-representative Gene Ontology (GO) terms of the differential expressed genes were determined using the Fisher’s exact test. The over-representative GO terms were depicted as a network via Cytoscape and EnrichmentMap.

### Knockout of C1GALT1 in MKN45 cells using CRISPR/Cas9 system

CRISPR/Cas9 system was used to knock out C1GALT1 in MKN45 cells. Small guide (sg) RNA for targeting C1GALT1 was designed according to database prediction (http://crispr.mit.edu/). The target sequence of sgC1GALT1 is 5′-GCAACACTTTGTTACAACGC-3′. Knockout of C1GALT1 in the genome was confirmed by DNA sequencing.

### In vivo xenograft animal models

For in vivo tumor growth analysis, 3 × 10^6^ of MKN45 cells or 10^7^ of AGS cells were mixed with Matrigel™ Basement Membrane Matrix (Corning Incorporated, Corning, NY, USA) and subcutaneously injected into five-week-old male nonobese diabetic/severe combined immunodeficiency (NOD/SCID) mice (National Laboratory Animal Center, Taiwan). AGS cells (2 × 10^6^) were used for tail vein injection, and the lungs were paraffin-embedded for hematoxylin and eosin (H&E) staining. For peritoneal injection, 10^7^ of MKN45 cells were used. Animal experiments were reviewed and approved by the Institutional Animal Care and Use Committee IACUC) of College of Medicine, National Taiwan University.

### Statistical analysis

Statistical analyses were performed using R 3.1.2 for Mac OS X and GraphPad Prism 6 for Mac OS X. The correlations between C1GALT1 expression and clinicopathologic characteristics were tested using a chi-square test. Student’s *t* test was used to compare differences between the two experimental groups. Univariate comparisons of the parameters were made using the nonparametric chi-square test or the Fisher and Mann–Whitney *U* tests. Overall survival was compared through log-rank comparisons for time-to-event data using Kaplan–Meier methods. Student’s *t* test was used to analyze in vitro and in vivo experiments. Two-sided *P* < 0.05 was considered statistically significant.

## Supplementary information

Supplementary Tables

Supplementary Figures
