# Supplementary material for: C1GALT1 is associated with poor survival and promotes soluble Ephrin A1-mediated cell migration through activation of EPHA2 in gastric cancer
Source: Oncogene. 2020 Jan 31;39(13):2724–40. doi: 10.1038/s41388-020-1178-7 (PMC7098884; doi:10.1038/s41388-020-1178-7)
Supplement: Supplementary file 1 — Supplementary Tables [file 41388_2020_1178_MOESM1_ESM.docx]

**Supplementary Table S1. Primers for PCR**

| **Gene** | **Primer sequence** | **Accession No.** |
| --- | --- | --- |
| *C1GALT1* | F: TGGGAGAAAAGGTTGACACC | NM_020156.4 |
|  | R: CTTTGACGTGTTTGGCCTTT |  |
| *CDK1* | F: CTGGGGTCAGCTCGTTACTC | NM_001786.4 |
|  | R: TCCACTTCTGGCCACACTTC |  |
| *KIF20A* | F: AGACAGCAAGCAGATCCGAC | NM_005733.2 |
|  | R: GGAGAAGCGAATGTTTGCCG |  |
| *KIF2C* | F: CCATGGACTCGTCGCTTCAG | NM_006845.3 |
|  | R: TGAGACAGTGGACATGCGAG |  |
| *BUB1* | F: CCAGGTGTACGAAGCTACCC | NM_004336.4 |
|  | R: CACAGTCATGCACTTGCTCA |  |
| *GBP1* | F: AGGGTCCAGTTGCTGAAAGAG | NM_002053.2 |
|  | R: TGGTACATGCCTTTCGTCGTC |  |
| *PRF1* | F: GACGTGACTCCTAAGCCCAC | NM_005041.4 |
|  | R: TGAAGTGGGTGCCGTAGTTG |  |
| *OAS2* | F: ACCGTTGGTGTTGGCATCTT | NM_016817.2 |
|  | R: TGTCTGCATTGTCGGCACTT |  |
| *BST2* | F: ATGGAAGACGGGGATAAGCG | NM_004335.3 |
|  | R: AGGAGATGGGTGACATTGCG |  |
| *GAPDH* | F: ACAGTCAGCCGCATCTTCTT | NM_002046.5 |
|  | R: GACAAGCTTCCCGTTCTCAG |  |

**Supplementary Table S2. Differential expression of selected genes in C1GALT1 knockdown AGS cells**

| **Functional pathway** | **gene** |
| --- | --- |
| Mitotic cell cycle | CCNB2, NUP54, CCND1, MAD2L1, CKS1B, CDK1, SPC25, PLK4, BIRC5, E2F5, NCAPG2, MIS12, BUB1, TOP2A, CENPW, PDS5B, NUF2, CCNA2, CCNB1, CDC20, RAB8A, SMC4, CASC5, AURKB, KIF20A, PTTG1, MCM5, NINL, VRK1, CDK6, SGO1, SKA1, KIF2C, SKP2, CENPE, UBE2C, CDCA5, LIN9, ARPP19, CDCA8, SGO2 |
| Cell cycle checkpoint | FOXO4, TTK, UBE2C, CCNG2, CDC20, MAD2L1, CDK1, BUB1, TOP2A, BIRC5, CCNA2, DTL, CLOCK, CASC5, AURKB, CCND1 |
| Microtubule-based movement | DYNC1I1, IFT81, DNAH9, SPAG16, DYX1C1, DNAH6, CCDC40, STK36, KIFC1, FYCO1, KIF4A, IFT140, KIF3A, KIF20A, BBS12, CCDC103, KIF2C, CENPE, MAP4, KIF14, RAB6A, DPCD |
| Regulation of cell division | MAD2L1, BIRC5, TGFA, ASPM, CIT, BUB1, PTTG3P, PTTG2, TTK, CCNB1, CDC20, POU5F1, AURKB, CHMP4C, PTTG1, PLCB1, CAT, CENPE, UBE2C, CENPV, FLCN, KIF14, EREG, CCSAP, MSX2, CDCA5 |
| Immune effector process | GBP3, PML, CFB, INPP5D, IFNE, GBP1, IRF1, IL33, IFIT3, TRIM22, BTN3A2, IFNB1, IFIT1, TLR3, NLRP3, IFNL3, C7, RNASEL, APOBEC3H, CDH17, POLR3G, PTPRC, OASL, ZC3HAV1, SERPING1, APOBEC3B, PTGER4, ITPR1, IFI44L, PRF1, RSAD2, RC3H2, MX2, SUSD4, C1RL, IFI16, OAS1, OAS2, TMEM173, CFI, STAT2, TLR4, SAMHD1, NLRC5, BST2, ATP7A, CTSH |

The differential expression genes validated by quantitative RT-PCR are highlighted.
