# Supplementary material for: C1GALT1 is associated with poor survival and promotes soluble Ephrin A1-mediated cell migration through activation of EPHA2 in gastric cancer
Source: Oncogene. 2020 Jan 31;39(13):2724–40. doi: 10.1038/s41388-020-1178-7 (PMC7098884; doi:10.1038/s41388-020-1178-7)
Supplement: Supplementary file 2 — Supplementary Figures [file 41388_2020_1178_MOESM2_ESM.pdf]

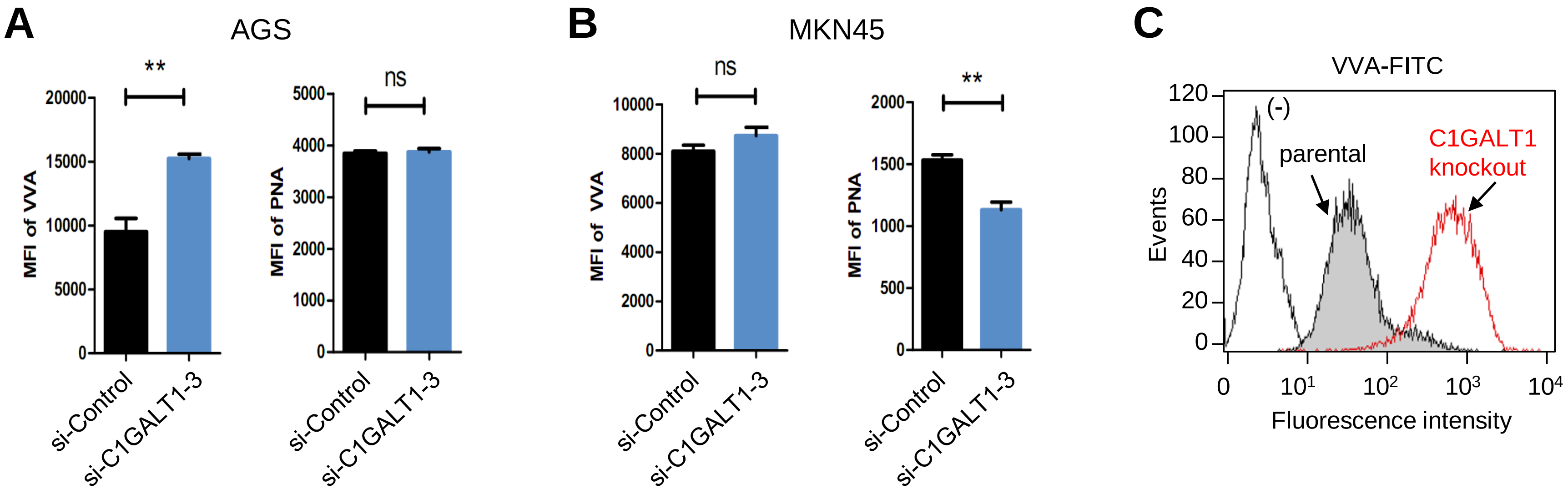

Supplementary Figure S1. C1GALT1 modulates surface O-glycans on gastric cancer cells. (A) C1GALT1 knockdown increased VVA binding to AGS cells. **(B)** C1GALT1 knockdown decreased PNA binding to MKN45 cells. C1GALT1 was knocked down with si-C1GALT1-3 for 3 days and then surface O-glycans were analyzed by flow cytometry with FITC-VVA and FITC-PNA. **$P < 0.01$; n = 3; ns, no significance. (**C**) Tn antigens were increased in C1GALT1 knockout MKN45 cells analyzed using flow cytometry with FITC-VVA. Statistical data were analyzed and obtained through Student's t-test and graphed as mean ± SD. **, $p < 0.01$.

Supplementary Figure S1

A

Reference											Reference
**EGFR**	**ErbB2**	ErbB3	ErbB4	FGFR1	FGFR2α	FGFR3	FGFR4	**Insulin R**	**IGF1R**	Axl	Dtk
Mer	**HGFR**	MSPR	PDGFRα	PDGFRβ	SCFR	**Flt-3**	MCSFR	C-Ret	ROR1	**ROR2**	Tie-1
Tie-2	TrkA	TrkB	TrkC	VEGFR1	VEGFR2	VEGFR3	MuSK	EphA1	EphA2	EphA3	EphA4
EphA6	EphA7	EphB1	EphB2	EphB4	EphB6	ALK	DDR1	DDR2	EphA5	EphA10	
Reference		EphB3	RYK								PBS

B

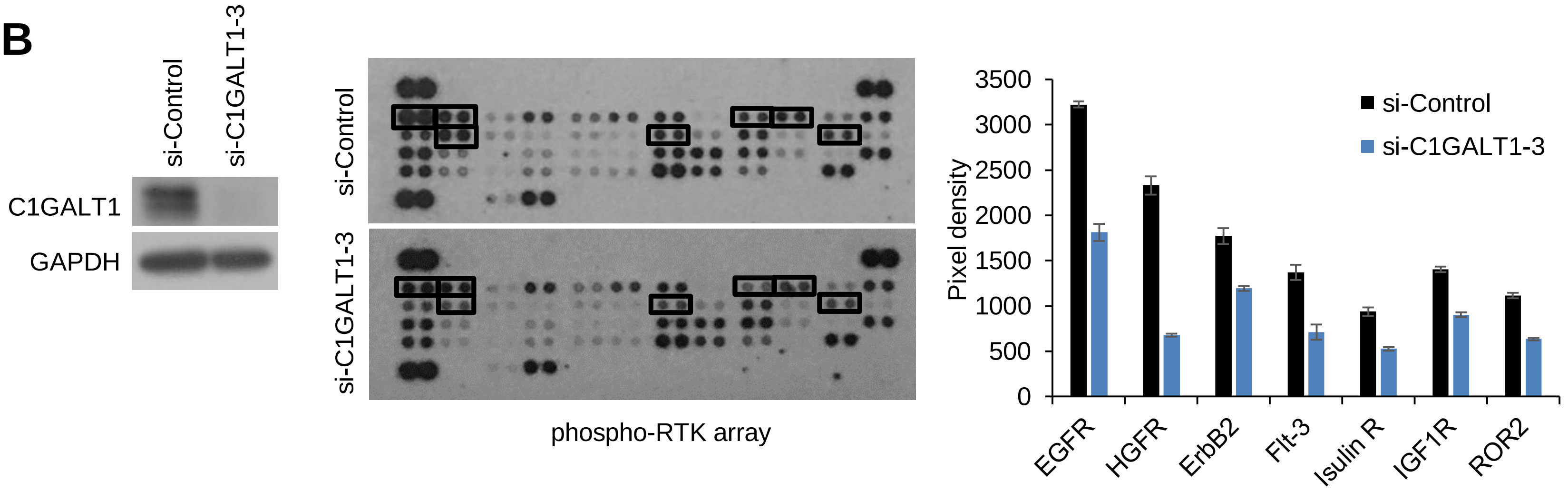

Supplementary Figure S2. Effects of C1GALT1 knockdown on multiple phospho-RTKs. (A) Template showing the location of 49 RTKs on the phospho-RTK array membrane. **(B)** Effects of C1GALT1 knockdown on the expression of phospho-RTKs in AGS cells. Cells were serum-starved for 8 h and subsequently treated with 10% FBS for 10 min. Phospho-RTKs in cell lysates were analyzed through Western blotting. C1GALT1 was knocked known with si-C1GALT1-3 (left panel). GAPDH was the loading control. Phospho-RTKs markedly decreased by si-C1GALT1-3 were boxed (middle panel). Quantification of signals for selected phospho-RTKs (right panel). pRTKs with pixel densities >500 and decreases >30% are shown. Pixel density was quantified using ImageJ.

Supplementary Figure S2

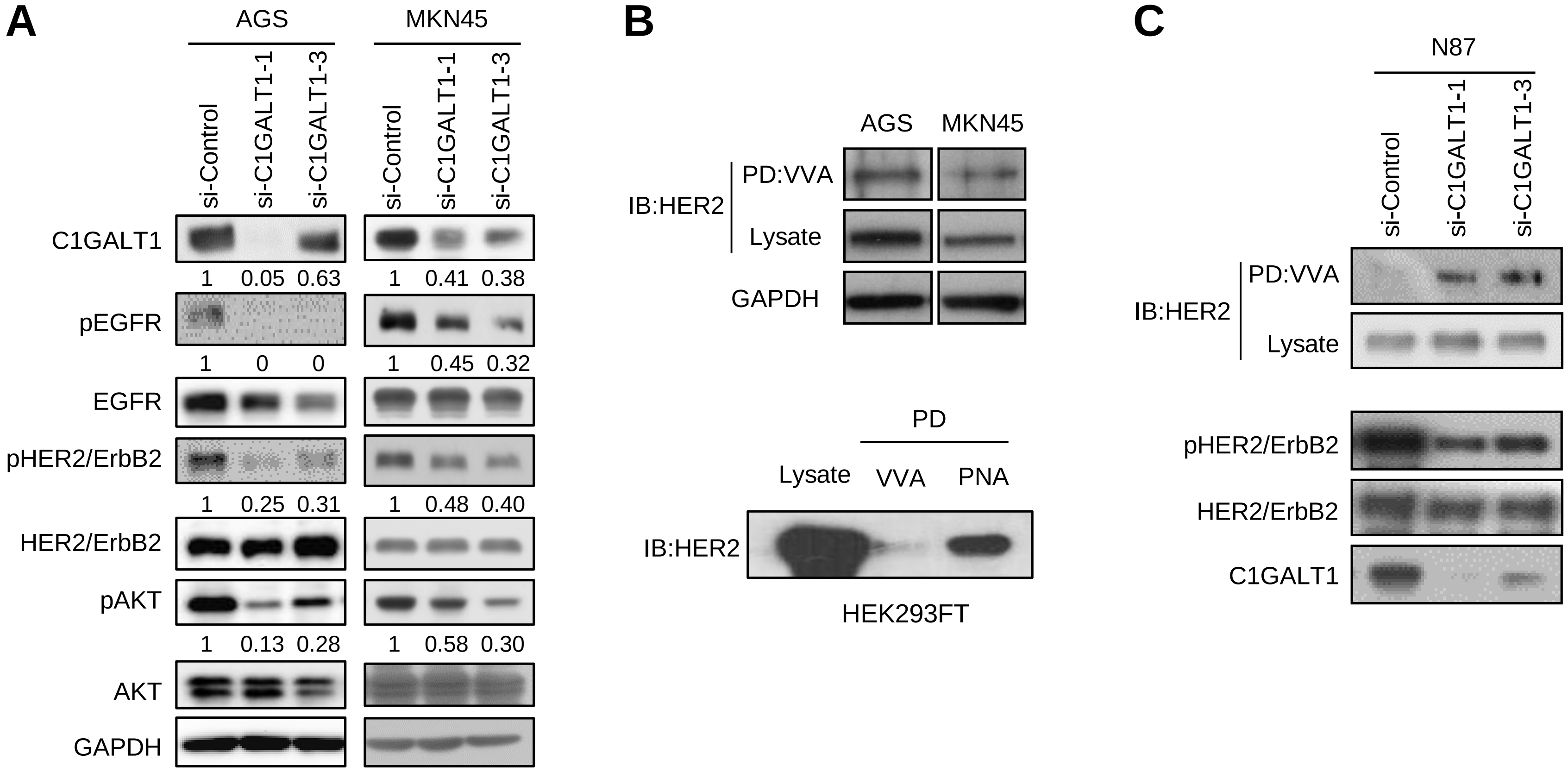

Supplementary Figure S3. C1GALT1 modifies phosphorylation and glycosylation of EGFR and HER2. (A) C1GALT1 knockdown inhibited phosphorylation of EGFR, HER2, and AKT. C1GALT1 was knocked down with si-C1GALT1-1 or si-C1GALT1-3 in AGS and MKN45 cells. Western blot analysis was performed to detect proteins as indicated. Band intensity was quantified using ImageJ and the relative protein levels are shown. A representative experiment from three independent experiments was shown. **(B)** HER2 was O-glycosylated. The presence of O-glycans on HER2 was demonstrated through lectin pull-down assays with VVA and PNA, which recognized GalNAc and Gal-GalNAc, respectively. AGS and MKN45 parental cells as well as HEK293FT cells overexpressing HER2 were used. To inhibit further elongation of O-glycans, HEK293FT cells were treated with 2 mM of benzyl-α-GalNAc (Sigma) for 48 h. PD, pull down; IB, immunoblotting. A representative experiment from three independent experiments was shown. **(C)** C1GALT1 knockdown modified O-glycans on HER2 and decreased phosphorylation of HER2 in N87 cells. C1GALT1 was knocked down with si-C1GALT1-1 or si-C1GALT1-3 in N87 cells. Upper panel, changes in O-glycans were analyzed through VVA pull-down assays. Cell lysates were treated with neuraminidase to remove sialic acids and then incubated with VVA beads. Lower panel, Western blots showing decreased phosphorylation of HER2 in C1GALT1 knockdown N87 cells. A representative experiment from three independent experiments was shown.

Supplementary Figure S3

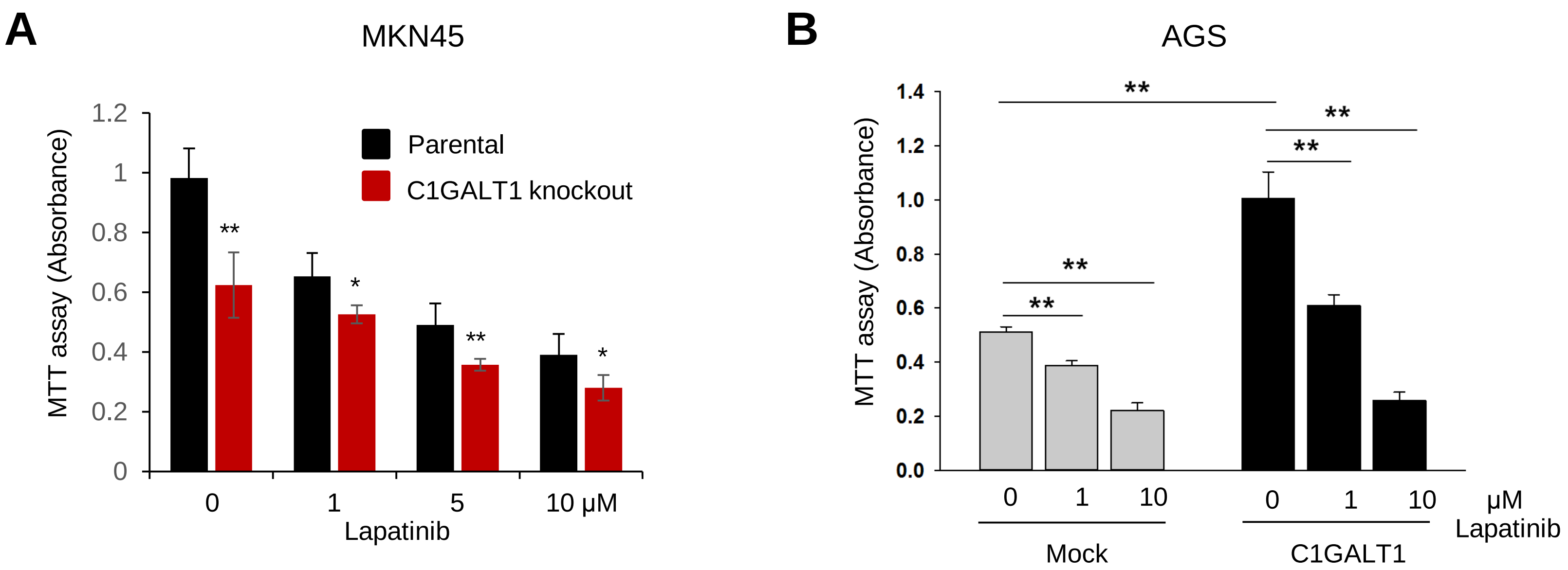

Supplementary Figure S4. C1GALT1-mediated cell growth is suppressed by lapatinib. (A) C1GALT1 knockout decreased cell viability in vitro. Cell viability of parental and C1GALT1 knockout MKN45 cells were analyzed by MTT assays at different concentrations of lapatinib, a dual inhibitor for EGFR and HER2 for 72 h. Data are represented as means ± SD from three independent experiments. *, $p < 0.05$; **, $p < 0.01$. **(B)** AGS cells were stably transfected with empty pcDNA3.1 or pcDNA3.1/C1GALT1 plasmid. Cell viability was analyzed by MTT assays. AGS cells were treated with DMSO control or lapatinib. MTT assays were performed after 72-h treatment. Statistic data are mean ± SD. Representative results of three independent experiments were shown. **$p < 0.01$ by student's *t*-test.

Supplementary Figure S4

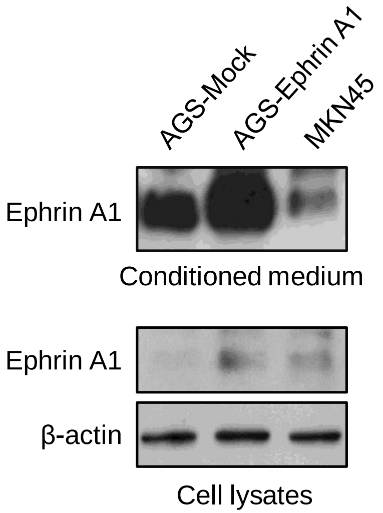

Supplementary Figure S5. Soluble Ephrin A1 is present in conditioned medium of gastric cancer cells. Ephrin A1 in conditioned medium and cell lysates in Mock and Ephrin A1 overexpressing AGS cells as well as parental MKN45 cells was analyzed by Western blotting. AGS cell were transfected with empty pcDNA3.1 plasmid (AGS-Mock) or pcDNA3.1-Ephrin A1 plasmid (AGS-Ephrin A1). Conditioned medium was collected from 24 h-cultured AGS and MKN45 cells in serum-free RPMI. β-actin was an internal control. A representative experiment from three independent experiments was shown.

Supplementary Figure S5

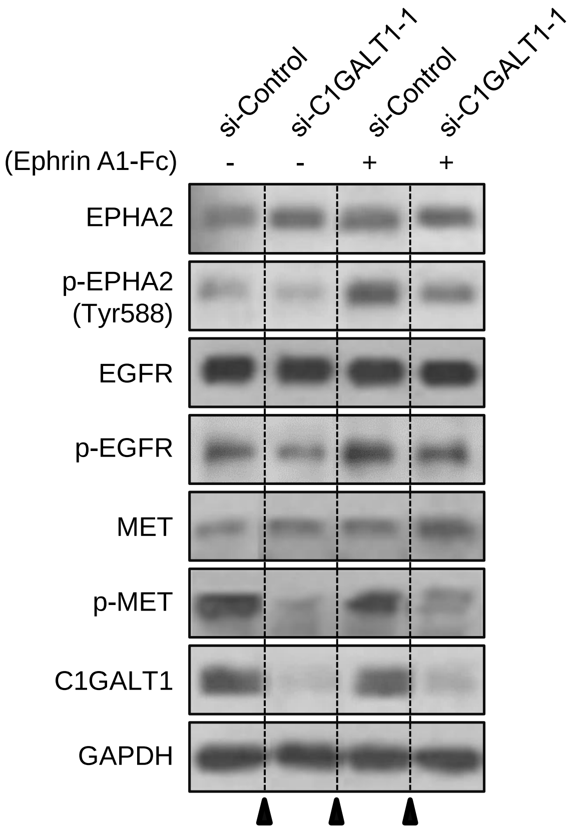

Supplementary Figure S6. C1GALT1 knockdown-mediated decrease in phosphorylation of EGFR and MET is not the downstream of the Ephrin A1-EPHA2 signaling pathway. AGS cells knocked down with si-Control or si-C1GALT1-1 were serum-starved for 24 h and then treated with or without Ephrin A1-Fc for 3 minutes. Phosphorylation of RTKs, as indicated, in cell lysates was analyzed using Western blotting. GAPDH was the loading control. Images were cut and rearranged from the same X-ray film. Arrowheads indicate the separation lines.

Supplementary Figure S6

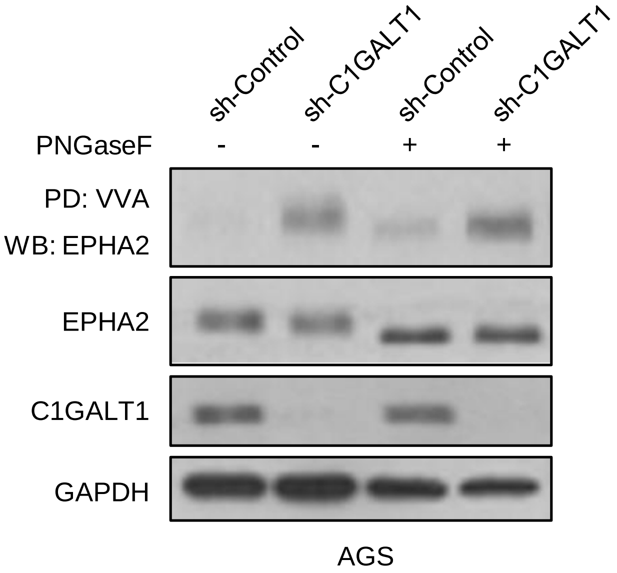

Supplementary Figure S7. C1GALT1 knockdown increases VVA binding to EPHA2 after removal of N-glycans. Changes in O-glycans on EPHA2 were analyzed through VVA pull-down assays. AGS cell lysates were treated with PNGaseF to remove N-glycans and then incubated with VVA beads. Western blots showing EPHA2 pulled down by VVA beads, input EPHA2, C1GALT1, and GAPDH. GAPDH was used as an internal control.

Supplementary Figure S7

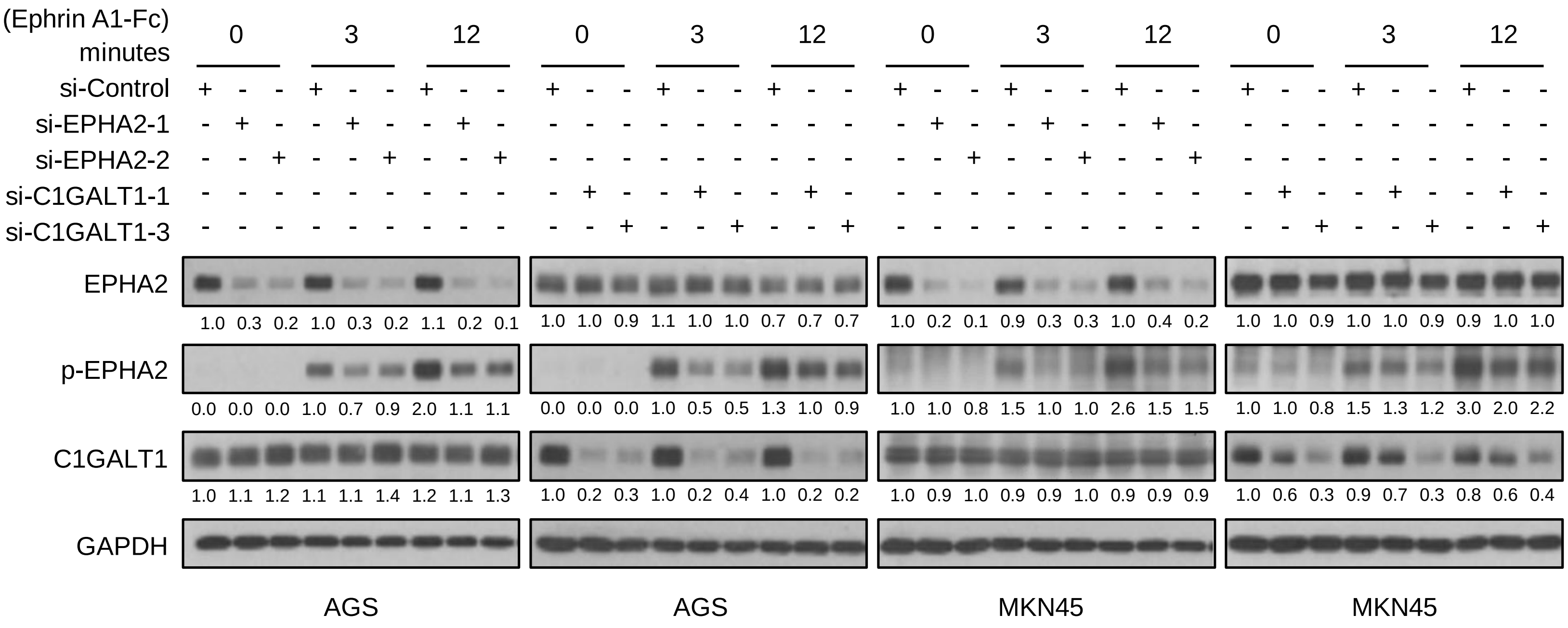

Supplementary Figure S8. Quantification of band intensities in Figure 6A. ImageJ was used to quantify protein signals of Western blots and relative intensities were shown. A representative experiment from three independent experiments was shown.

Supplementary Figure S8

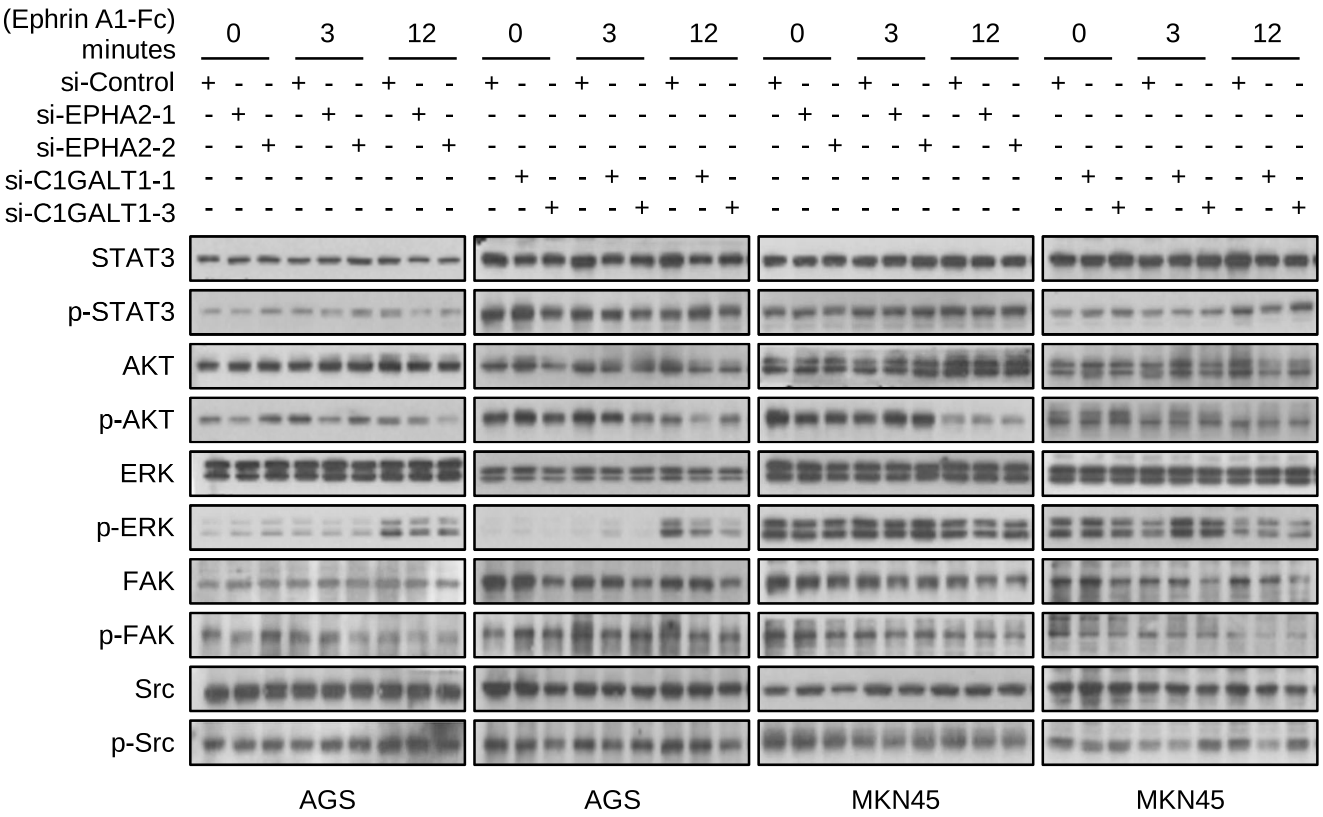

Supplementary Figure S9. Impacts of C1GALT1 or EPHA2 knockdown on signaling in gastric cancer cells. EPHA2 or C1GALT1 knockdown cells were treated with Ephrin A1-Fc, as indicated, and cell signaling was analyzed by Western blotting. Notably, ERK phosphorylation was induced by Ephrin A1-Fc in AGS cells. A representative experiment from three independent experiments was shown.

Supplementary Figure S9

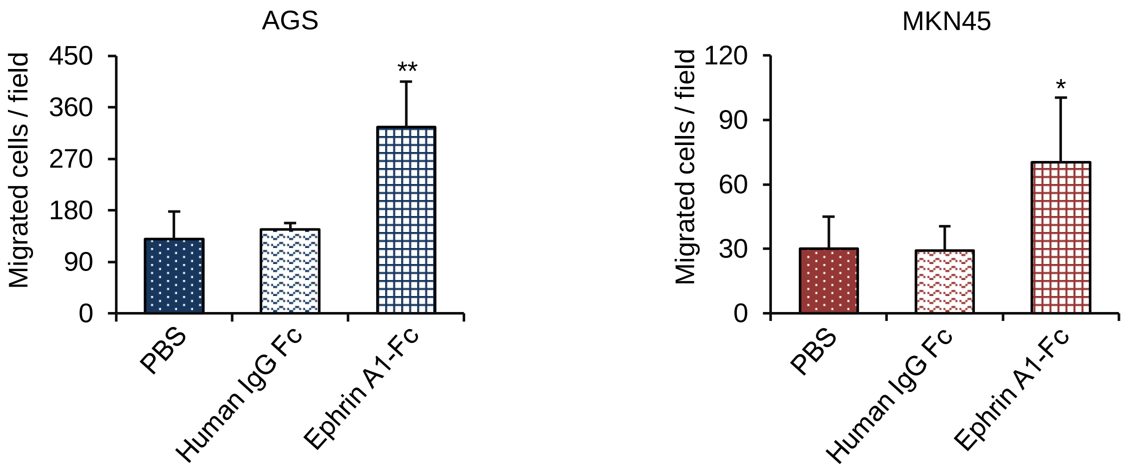

Supplementary Figure S10. Human IgG Fc does not trigger EPHA2 migration in gastric cancer cells. AGS and MKN45 cells were pretreated with PBS, human IgG Fc or Ephrin A1-Fc respectively. After that, 10% FBS-triggered migration was analyzed using transwell migration assays. **, $P < 0.01$. *, $P < 0.05$.

Supplementary Figure S10

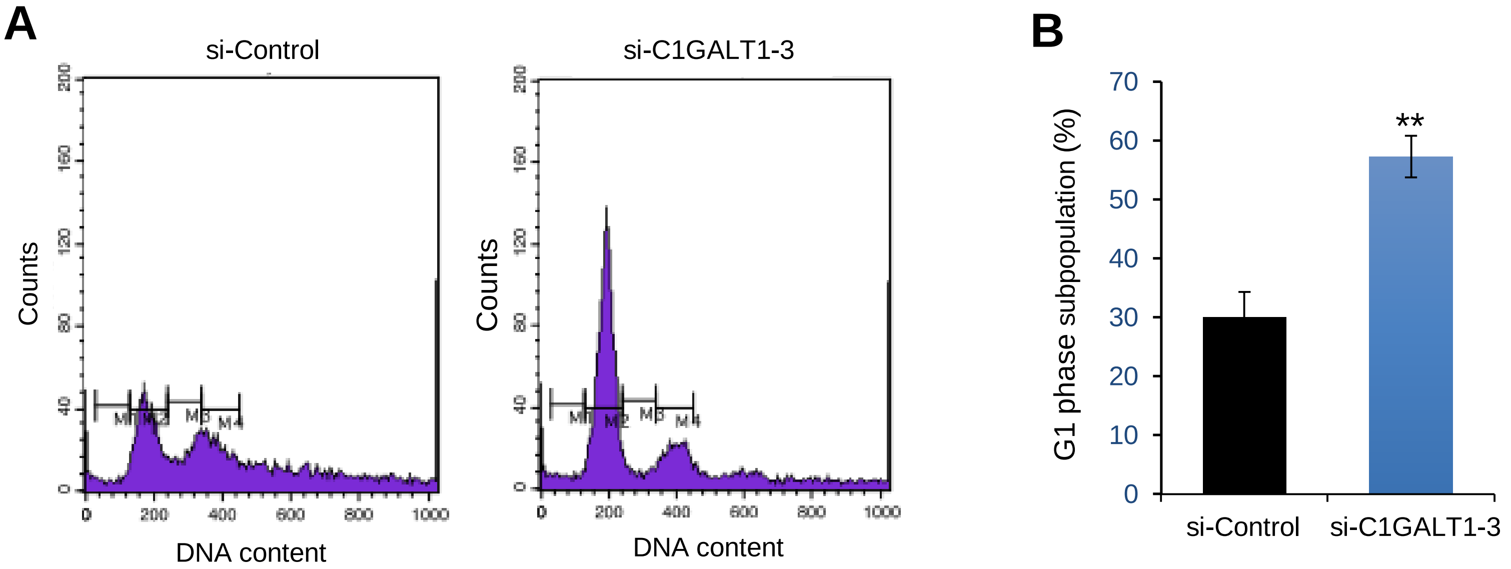

Supplementary Figure S11. C1GALT1 knockdown causes cell cycle arrest in gastric cancer cells. (A) Cell cycle analysis. C1GALT1 was knocked down with si-C1GALT1-3 in AGS cells and DNA content was analyzed by flow cytometry with propidium iodide (PI). M2 indicates G1 phase subpopulation. Non-targeting siRNA (si-Control) was used for control. Representative results are shown. **(B)** C1GALT1 induced cell cycle arrest at G1 phase. Statistic data are mean ± SD of three independent experiments. $^{**}p < 0.01$ by student's *t*-test.

Supplementary Figure S11
